# Curcumin Modulates PTPRZ1 Activity and RNA m6A Modifications in Neuroinflammation‐Associated Microglial Response

**DOI:** 10.1002/advs.202405263

**Published:** 2025-02-08

**Authors:** Ninan Zhang, Ruifan Lin, Wenya Gao, Honglin Xu, Yuejia Li, Xiahe Huang, Yingchun Wang, Xianghong Jing, Wenxiang Meng, Qi Xie

**Affiliations:** ^1^ Institute of Acupuncture and Moxibustion China Academy of Chinese Medical Sciences Beijing 100700 China; ^2^ Institute of Basic Research in Clinical Medicine China Academy of Chinese Medical Sciences Beijing 100700 China; ^3^ State Key Laboratory of Molecular Developmental Biology Institute of Genetics and Developmental Biology Chinese Academy of Sciences Beijing 10019 China; ^4^ University of Chinese Academy of Sciences Beijing 100049 China; ^5^ Innovation Academy for Seed Design Chinese Academy of Sciences Beijing 100101 China; ^6^ Wangjing Hospital of China Academy of Chinese Medical Sciences Beijing 100102 China

**Keywords:** anti‐inflammatory, curcumin, m6A post‐transcriptional modifications of RNA, microglia homeostasis, PTPRZ1

## Abstract

Neuroinflammation is often characterized by an overactive microglial response. Curcumin, known for its anti‐inflammatory and antioxidant properties, can mitigate microglial hyperactivity following epileptic seizures. The study delves into the molecular mechanisms underlying curcumin's modulation of RNA post‐transcriptional N (6)‐methyladenosine (m6A) modification. It is found that curcumin interacts with the Z1‐type protein tyrosine phosphatase receptor (PTPRZ1), maintaining its enzymatic activity and thus regulating the phosphorylation of the m6A‐reader YTH domain‐containing family protein 2 (YTHDF2). This modulation affects the expression of critical genes, resulting in reduced inflammatory responses. These findings highlight the importance of post‐transcriptional modifications of RNA in the neuroprotective and anti‐inflammatory effects of curcumin, offering new insights for the treatment of related diseases.

## Graphical abstract

Wenxiang Meng and colleagues delve into the molecular mechanisms underlying curcumin's modulation of RNA post‐transcriptional N (6)‐methyladenosine (m6A) modification. It is found that curcumin interacts with the Z1‐type protein tyrosine phosphatase receptor (PTPRZ1), maintaining its enzymatic activity and thus regulating the phosphorylation of the m6A‐reader protein YTHDF2. This modulation affects the expression of critical genes, resulting in reduced inflammatory responses.

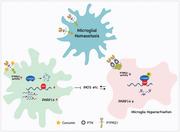

## 1 Introduction

Neuroinflammation is a central process in the brain's response to injury or disease, with microglia serving as the primary drivers. These cells are not only vital components of the brain's environment but also play a pivotal role in maintaining the balance of the central nervous system (CNS).^[^ ^1^ ^]^ The activity of microglia is closely associated with the progression and outcomes of neurological conditions such as epilepsy, stroke, and Parkinson's disease.^[^ ^1^, ^2^ ^]^ In epilepsy, the hyperresponsiveness of microglia can alter the extraneuronal environment, exacerbating symptoms.^[^ ^3^ ^]^ However, an appropriate response can have the opposite effect, by limiting seizures, reducing the excessive release of inflammatory factors, and aiding in the repair and regeneration of damaged neurons. Thus, a proper microglial response can positively influence epileptogenesis by limiting CNS damage and promoting post‐seizure recovery, making it a target of clinical exploration.^[^ ^4^ ^]^

Curcumin, a traditional bioactive compound, has been used for centuries in India and China to address various health conditions, including epilepsy.^[^ ^5^ ^]^ Contemporary research suggests that supplementation with curcumin may potentially mitigate seizure symptoms by modulating the balance of the central nervous system's microenvironment, thanks to its anti‐inflammatory and antioxidant properties.^[^ ^6^ ^]^ Despite extensive studies into the molecular mechanisms of curcumin, particularly its influence on the expression of various inflammatory factors, the precise direct targets and intricate molecular mechanisms underpinning curcumin's effects largely remain a mystery.^[^ ^7^ ^]^

To explore these mechanisms, we employ a combination of phosphoproteomics, MeRIP‐seq, and RNA‐seq analyses to investigate how curcumin interacts with inflammatory signaling pathways post‐status epilepticus. We found that curcumin directly targets the Z1‐type protein tyrosine phosphatase receptor (PTPRZ1), maintaining its enzymatic activity. This interaction significantly affects the phosphorylation state of the m6A reader protein YTH domain containing family protein 2 (YTHDF2), subsequently altering gene expression profiles that are crucial in regulating microglial activity and inflammatory responses. These results not only underscore the potent anti‐inflammatory and neuroprotective effects of curcumin but also highlight its potential as a therapeutic agent in the management of neuroinflammatory diseases.

## 2 Results

### 2.1 Curcumin Directly Interacts with PTPRZ1 and Maintains its Activity

**Figure 1:**
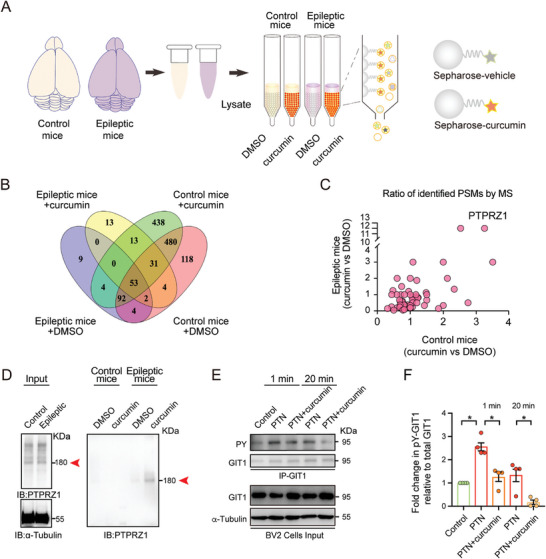
Affinity chromatography for curcumin target identification. A) Scheme of affinity chromatography. B) Venn diagram displaying four groups of mass spectrometry data C) Among the 53 candidate targets, PTPRZ1 exhibited a higher binding affinity for curcumin in epileptic mice than in control mice. D) Western blot results showed that PTPRZ1 strongly interacted with curcumin after status epilepticus. E) BV2 cells were treated with PTN (100 nm) alone, PTN combined with curcumin (20 µm), or DMSO (control) for 1 minute and 20 min. Immunoprecipitation (IP) was performed to assess tyrosine phosphorylation (PY) levels of GIT1. Western blotting shows the IP samples of phosphorylated GIT1 (IB: PY) and total GIT1 levels (IB: GIT1). The input panel shows total GIT1 and α‐Tubulin as a loading control in BV2 cell lysates. F) Quantification of phosphorylated GIT1 protein levels. Data are the mean ± SEM. from four independent experiments. *p < 0.05, by Student's *t*‐test.

To assess how curcumin influences microglial stability, we utilized a pilocarpine‐induced status epilepticus (SE) mouse model, closely replicating the transition from isolated seizures to chronic epilepsy and reflecting human inflammatory responses and seizure patterns.^[^ ^8^ ^]^ Through affinity chromatography coupled with mass spectrometry, we identified proteins that bind specifically to curcumin in the brain tissue of epileptic mice (**Figure** **1**A, Table S1, Supporting Information). This approach enabled us to pinpoint 53 proteins that showed enhanced binding affinity to curcumin under disease conditions, facilitating precise protein quantification (Figure 1B and Figure S1A, Supporting Information). In our studies, we observed that PTPRZ1 displayed a pronounced affinity for curcumin in whole‐cell lysates derived from the brains of epileptic mice (Figure 1C and Figure S1B, Supporting Information), a finding we corroborated through Western blot analysis (Figure 1D). This finding is significant, confirming PTPRZ1 as a direct target of curcumin and underscoring its potential role in modulating the effects of curcumin on neuronal and glial populations, particularly microglia.

PTPRZ1, also known as RPTPβ and RPTPζ, is a key component of the receptor‐type protein tyrosine phosphatase family, which plays a crucial role in regulating cellular processes such as proliferation, adhesion, and migration by dephosphorylating its substrates.^[^ ^9^ ^]^ To determine the impact of curcumin on the enzymatic activity of PTPRZ1, we utilized BV2 cells, a microglia‐like murine glial cell line, which serves as a model to mimic immune responses in the CNS.^[^ ^10^ ^]^ These cells were treated with Pleiotrophin (PTN) both alone and in combination with curcumin (Figure 1E). PTN, a significant neurotrophic factor and a natural inhibitory ligand for PTPRZ1, is known to be upregulated during epileptic activities and in activated microglia.^[^ ^11^ ^]^ Our results indicated that curcumin significantly attenuates PTN‐induced phosphorylation of G protein coupled receptor kinase interacting protein 1 (GIT1), a well‐established substrate of PTPRZ1,^[^ ^12^ ^]^ suggesting that curcumin effectively maintains the enzymatic action of PTPRZ1 under neuroinflammatory conditions (Figure 1F). This finding shows that curcumin and PTPRZ1 interact directly, and this may be linked to neuroinflammation.

**Figure 2:**
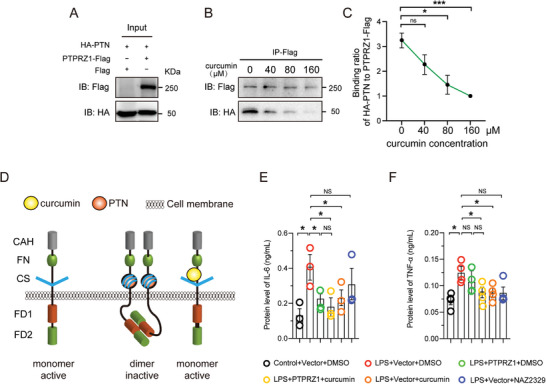
PTPRZ1 as a functional target of curcumin. A,B) Co‐immunoprecipitation of HA‐PTN and PTPRZ1‐Flag (or pCAH‐Flag) following overexpression. The same lysate was used for all curcumin concentration groups (0, 40, 80, and 160 µm) to ensure consistent HA‐PTN and Flag‐PTPRZ1 expression across conditions. C) Quantification of HA relative to PTPRZ1‐Flag after exposure to different curcumin concentrations (*n =* 3 biologically independent experiments. *p < 0.05; ***p < 0.001; ns (not significant), by Student's *t*‐test. D) Schematic diagram of curcumin maintaining PTPRZ1 activity by competitively blocking PTN. E,F) Elisa analysis of protein levels of IL‐6 and TNF‐α in BV2 cells overexpressing PTPRZ1‐Flag or pCAH‐Flag for 24 h, followed by treatment with LPS (250 ng mL^−1^), curcumin (10 µm), NAZ2329 (25 µm), or vehicle (control) for 16 h in serum‐free medium. Data are mean ± SEM., *n =* 3 or 4 biologically independent experiments by Student's *t*‐test.

Previous research indicated that PTN can respond to neuroinflammatory stress by binding to the extracellular domain of PTPRZ1, thereby inhibiting its activity.^[^ ^13^ ^]^ We further explored this interaction under conditions influenced by curcumin. Utilizing co‐immunoprecipitation techniques in HEK 293T cells engineered to express Flag‐tagged PTPRZ1 and HA‐tagged PTN, we systematically varied the concentrations of curcumin to assess its impact on this interaction (**Figure** **2**A,B). Our results demonstrated a dose‐dependent decrease in the binding affinity between PTPRZ1 and PTN with increasing concentrations of curcumin (Figure 2B,C). This observation suggests that curcumin may disrupt the PTN‐PTPRZ1 interaction (Figure 2D), potentially altering the downstream signaling pathways involved in neuroinflammatory responses. Importantly, additional experiments confirmed that curcumin does not directly bind to PTN (Figure S2A, Supporting Information), implying that curcumin may exert its modulatory effects on the PTN‐PTPRZ1 complex. Given the complexity of these interactions and their potential implications, further in vivo studies are essential to validate these findings and fully elucidate the therapeutic potential of curcumin in modulating neuroinflammatory pathways.

### 2.2 Curcumin Modulates LPS‐Induced Microglial Responses Potentially via PTPRZ1

Curcumin's modulatory effect on microglial response has been well‐documented.^[^ ^14^ ^]^ To investigate its role in neuroinflammation, we utilized the Lipopolysaccharide (LPS) model, which reliably induces immune responses analogous to those in neuroinflammatory conditions, such as epilepsy.^[^ ^15^ ^]^ The LPS model is pertinent as it replicates the key features of microglial activation observed in vivo, providing a valid platform for elucidating curcumin's effects under neuroinflammatory conditions.

To further explore the interaction between PTPRZ1 and curcumin in modulating neuroinflammatory responses, we transfected BV2 cells with either a PTPRZ1‐expressing vector or a control vector and subsequently exposed them to LPS to induce inflammation, which resulted in reduced levels of key inflammatory cytokines like interleukin 6 (IL‐6) and interleukin 1 beta (IL1‐β) (Figure 2E and Figure S2B, Supporting Information). However, tumor necrosis factor alpha TNF‐α) levels remained unchanged (Figure 2F), suggesting a selective regulation of inflammatory factors by PTPRZ1. Interestingly, PTPRZ1 overexpression alone reduced the release of the above cytokines, similar to the effect of curcumin alone, while the combination of PTPRZ1 and curcumin did not lead to further decreases (Figure 2E). This observation implies that curcumin may share similar mechanisms with PTPRZ1 in modulating inflammatory responses, highlighting the potential role of PTPRZ1 in mediating the effect of curcumin on neuroinflammation.

### 2.3 Identification of Substrate Regulated by Curcumin‐PTPRZ1 Interaction

There is an abundance of evidence indicating that hyperactivation of protein kinases plays a crucial role in activating microglial cells, while phosphatases such as PTPRZ1 and its ligand PTN, which tend to increase during this process, have opposing effects.^[^ ^16^ ^]^ Our data indicate that both PTPRZ1 and curcumin effectively suppress LPS‐induced cytokine expression (Figure 2E,F; Figure S2B, Supporting Information), underscoring the significant role of PTPRZ1 in regulating microglial responses.

**Figure 3:**
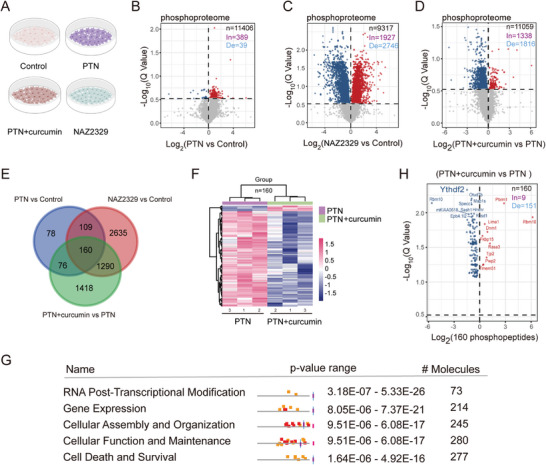
YTHDF2 has been identified as a pivotal substrate regulated by PTPRZ1 through phosphorylation mass spectrometry analysis. A) Schematic diagram of phosphorylation mass spectrum groups. BV2 cells were incubated with PTN (100 nm), curcumin (20 µm), NAZ2329 (10 µm), or vehicle (control) for 20 min before processing for mass spectroscopy. B–D) Volcano plots show changes in phosphopeptides in PTN versus control, NAZ2329 versus control, and PTN+curcumin versus PTN. Multiple Student's *t*‐test (*P* value) was followed by false discovery rate (FDR) (*Q* value) analysis. In, increase; De, decrease. E) Venn diagram of significantly changed phosphopeptides among the three experimental groups, with the number of significantly changed phosphopeptides in each experiment. F) Cluster analysis of 160 phosphopeptides that are changed in PTN and PTN with curcumin groups. G) The bioinformatics analysis by IPA used 160 phosphopeptides and their sites. H) Volcano plots showing YTHDF2 is the most likely substrate of PTPRZ1 in the PTN+curcumin versus PTN group among 160 phosphopeptides.

To elucidate the downstream effects triggered by the binding of curcumin to PTPRZ1, BV2 cells were treated with DMSO, PTN, a combination of curcumin and PTN, and the PTPRZ1 inhibitor NAZ2329 for 20 min. We then performed a phosphorylation mass spectrometry analysis on the whole cell lysates to assess changes in protein phosphorylation levels (**Figure** **3**A and Table S2, Supporting Information). Both PTN and NAZ2329 are known to bind specifically to PTPRZ1 and inhibit its activity.^[^ ^17^ ^]^ The analysis revealed that PTN and NAZ2329 treatment led to increased phosphorylation levels of specific peptides, affirming the role of PTPRZ1 as a tyrosine phosphatase (Figure 3B,C). Conversely, curcumin was observed to mitigate the inhibitory effects of PTN on PTPRZ1, with a significant decrease in phosphorylation, with only approximately 58% (1816/3154) of the phosphorylated peptides (Figure 3D). This observation suggests a strong inhibitory influence of curcumin on PTN‐mediated phosphorylation of PTPRZ1 substrates, reinforcing our hypothesis that curcumin actively antagonizes the PTN‐induced inhibition of PTPRZ1.

We focused on the downstream targets of PTPRZ1 and identified 160 phosphopeptides whose average intensity showed significant changes in three experiments (PTN versus control, NAZ2329 versus control, curcumin with PTN versus PTN) (Figure 3E,F). Our cluster analysis revealed that curcumin can reverse the inhibitory effect of PTN on PTPRZ1 and promote the remodeling of the cellular phosphoproteome (Figure 3F). Furthermore, bioinformatics analysis of the 160 identified phosphopeptides revealed their involvement in pathways crucial for RNA processing and gene expression regulation (Figure 3G), suggesting a comprehensive role of curcumin in modulating microglial activation by influencing these key molecular pathways.

### 2.4 Curcumin Modulates RNA m6A Modification through YTHDF2 Phosphorylation Mediated by PTPRZ1

Notably, unlike PTN alone, the level of phosphorylation of the key m6A reader protein YTHDF2 was significantly reduced when curcumin was combined with PTN (Figure 3H). YTHDF2's primary role is to regulate the stability of m6A‐modified mRNA within the cytoplasm, potentially impacting demethylation processes within the nucleus.^[^ ^18^ ^]^ This underscores the significant role that RNA m6A modification plays in regulating the cellular environment and homeostasis. Our findings suggest that curcumin's modulation of YTHDF2 phosphorylation is an essential mechanism through which curcumin can influence these crucial biological processes.^[^ ^1^, ^19^ ^]^

**Figure 4:**
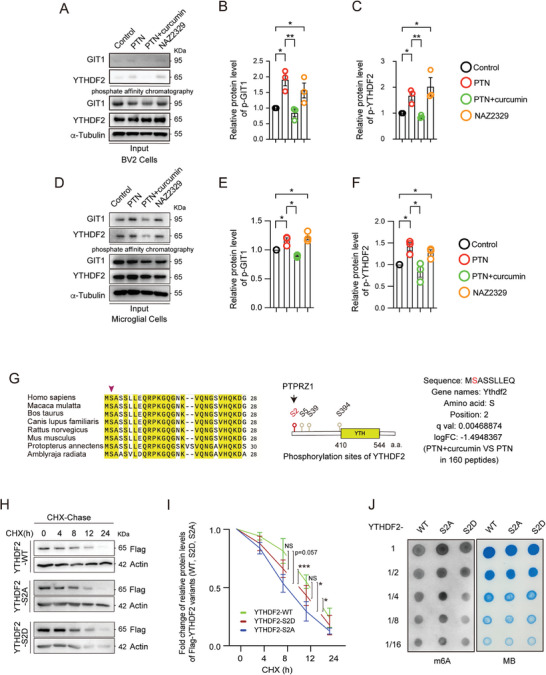
Curcumin modulates the stability of YTHDF2 via dephosphorylation of the S2 site and subsequently affects the level of RNA m6A modification. A) Phosphate affinity chromatography using Phos‐tag™ agarose in Bv2 cells subjected to various conditions: control, PTN, PTN+curcumin, and NAZ2329. GIT1 is used as a positive control for the PTPRZ1 substrate. The upper panels show the phosphorylated forms of GIT1 and YTHDF2, while the lower panels display total GIT1, YTHDF2, and α‐Tubulin (loading control) in the input samples. B,C) Phosphorylated protein quantification of GIT1 and YTHDF2 from (A). Data are the mean ± SEM. *n =* 3 biologically independent experiments. *p < 0.05; **p < 0.01 by Student's *t*‐test. D) Phosphate Affinity Chromatography using Phos‐tag^TM^ agarose in primary microglia cells subjected to various conditions. E,F) Phosphorylated protein quantification of GIT1 and YTHDF2 from (D). Data are the mean ± SEM. *n =* 3 biologically independent experiments. *p < 0.05 by Student's *t*‐test. G) The S2 site of YTHDF2 is highly conserved between species (left panel) and a schematic representation of YTHDF2 phosphorylation sites (middle panel) regulated by PTPRZ1. The phosphorylation mass spectrometry data for YTHDF2 compare PTN+curcumin to PTN treatment (right panel). H) The stability of wild‐type (WT) or S2A or S2D mutated Flag‐YTHDF2 was measured by CHX (100 µg mL^−1^) chase assay in 293T cells, CHX (Cycloheximide). I) Analysis of YTHDF2 protein lifetime of (H). Data are mean ± SEM., *n =* 3 biologically independent experiments. *p < 0.05, ***p < 0.001 by Student's *t*‐test. J) Dot blot was used to detect the m6A modification after overexpression of YTHDF2‐WT, YTHDF2‐S2A, and YTHDF2‐S2D in BV2 cells. Methylene blue staining was used as a loading control (*n =* 3), MB (Methylene blue).

To validate the phosphoproteomic findings, primary microglia (Figure S3A,B, Supporting Information) and BV2 cells were treated with PTN, PTN combined with curcumin, and the PTPRZ1 inhibitor NAZ2329. Phosphate affinity chromatography was utilized to enrich phosphorylated proteins (**Figure** **4**A–F). The results indicated that YTHDF2, like GIT1 and p190 RhoGAP, the known substrates of PTPRZ1, exhibited hyperphosphorylation in the groups treated with PTN and NAZ2329, compared to the control (Figure 4B,C,E,F and Figure S3C, Supporting Information). Conversely, curcumin treatment significantly mitigated the hyperphosphorylation of YTHDF2, GIT1, and p190 RhoGAP in both types of PTN‐treated cells. These findings suggest that curcumin can modulate the phosphorylation of YTHDF2 in microglial cells.

Previous studies have established that the phosphorylation of YTHDF2 affects its stability.^[^ ^20^ ^]^ Our phosphoproteomics data indicated that the second serine (S2) of YTHDF2 undergoes significant changes after curcumin administration (Figure 4G). To examine the function of this phosphorylation, we replaced serine (S) with alanine (A) or asparanin (D) to mimic corresponding non‐phosphorylated or phosphorylated states. We transfected the constructs into HEK 293T cells and conducted cycloheximide (CHX) chase experiments to assess the stability of the expressed proteins (Figure 4H). CHX is used to inhibit protein synthesis, allowing us to measure the degradation rate of the protein over time, thereby providing insights into protein stability.^[^ ^21^ ^]^ As expected, the phosphorylated mutant (S2D) was more stable than the non‐phosphorylated mutant (S2A) in samples collected at the 8 h and 12 h time point during CHX chase (Figure 4I). Our findings suggest the phosphorylation of YTHDF2 at S2 affects its stability.

YTHDF2, as a key “reader” of m6A‐modified mRNA, is crucial for maintaining RNA homeostasis. To verify the effects of phosphorylation at the S2 site of YTHDF2, we conducted functional assays following a mutation at this site (Figure 4J). Dot blot analysis of total RNA from cells expressing the non‐phosphorylated mutant YTHDF2‐S2A revealed a significant increase in m6A modification, implicating the phosphorylation state of S2 in regulating overall mRNA m6A modifications due to the increased degradation of YTHDF2 (Figure 4H–J).

### 2.5 Curcumin Regulates RNA m6A Modification and Affects Gene Expression

**Figure 5:**
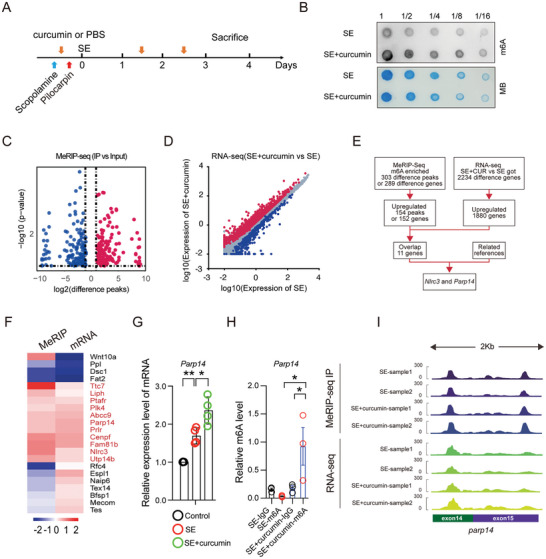
Curcumin upregulates *Parp14* mRNA m6A and mRNA expression after status epilepticus. A) Experimental timeline for SE, SE + curcumin mice. B) A dot blot was used to detect the m6A modification in SE and SE + curcumin mice. Methylene blue staining was used as a loading control (*n =* 3). C) Volcano plots showing difference peaks in MeRIP‐seq, Student's *t*‐test (P value) analysis. Red represents an increase; blue represents a decrease. D) RNA‐seq showing the total gene expression in SE+curcumin versus SE. Red represents increase; blue represents decrease. (Student's *t*‐test, *P* < 0.05). E) Schematic workflow of MeRIP‐seq combined with RNA‐seq data analysis stream. F) Cluster analysis of genes identified by MeRIP‐seq and RNA‐seq analyses, of which 11 are upregulated in both expression and mRNA m6A modification. Red represents an increase; blue represents a decrease. G) Analysis of *Parp*14 mRNA levels in control, SE, and SE+curcumin mice groups. Data are mean ± SEM., *n =* 4 by Student's *t*‐test. H) m6A enrichment in *Parp*14 between SE and SE+curcumin groups by MeRIP‐qPCR. I) Integrative Genomics Viewer (IGV) tracks displaying MeRIP‐seq (upper panels) and RNA‐seq (lower panels) read distribution in *Parp*14 mRNA of SE and SE+curcumin mice.

Curcumin's impact on the phosphorylation status of YTHDF2 implicates it in modulating mRNA m6A modification and gene expression. To investigate this hypothesis, we conducted an in vivo study using a status epilepticus (SE) model in mice to assess how curcumin affects RNA m6A modification (**Figure** **5**A). Our findings revealed that curcumin treatment significantly increased m6A modification levels in total RNA extracted from the hippocampus (Figure 5B), indicating tissue‐specific modulation of m6A modification by curcumin.

To examine the potential impact of this modification on gene expression, we performed combined MeRIP‐Seq and RNA‐Seq analyses to analyze the distribution of m6A modifications and the corresponding changes in gene expression within the hippocampus (Figure 5C,D; Tables S3 and S4, Supporting Information). Our MeRIP‐Seq data showed significant enrichment of RNA m6A peaks, particularly in RGACH motifs (R = G/A; H = A/C/U), which are critical in regulating mRNA stability (Figure S4A, Supporting Information). These peaks were predominantly located in the coding sequences (CDS), 3′ untranslated regions (UTR), and near‐stop codons. This distribution confirms our understanding of the role of m6A in influencing mRNA translation and stability, thereby validating our MeRIP sequencing approach (Figure S4B,C, Supporting Information).

From the MeRIP‐Seq analysis, we identified 303 differential peaks (Figure 5C,E), with 154 peaks notably upregulated solely in the curcumin‐treated group. This pattern suggests that curcumin can intricately modulate the transcriptome through m6A‐dependent mechanisms. Moreover, our RNA‐Seq analysis identified 2234 distinct genes, with 1880 showing upregulation, highlighting curcumin's substantial influence on gene expression patterns in the hippocampus (Figure 5D,E).

Further bioinformatics analysis linked these gene expression changes to biological processes associated with the inflammatory response (Figure S5, Supporting Information), aligning with the observed effects of curcumin on microglial activation. These findings not only underscore curcumin's capacity to influence gene expression through m6A modifications but also enhance our understanding of its potential therapeutic impact on neuroinflammatory conditions.

### 2.6 Parp14 as a Regulatory Effector of YTHDF2

Our objective was to find genes that regulate the inflammatory response (Figure S5, Supporting Information). To achieve this, we observed changes in mRNA levels and enrichment of RNA m6A modification in epileptic mice treated with curcumin (Figure 5E,F). We found 11 genes that were consistent with the expectation that both the total amount of RNA and the m6A modification level would increase after curcumin administration, and among these, poly ADP ribosyl polymerase 14 (Parp14) and NOD like receptor family CARD domain containing 3 (Nlrc3) are reported to be highly associated with the regulation of the inflammatory response.^[^ ^22^ ^]^

To confirm the outcome, we isolated mRNA from the hippocampus and cortex of SE mice and curcumin‐treatment SE mice, respectively. We then utilized MeRIP‐IP to concentrate m6A‐modified mRNA. Results showed that the total quantity of Parp14 and Nlrc3 mRNA, as well as the m6A modification, was increased significantly (Figure 5G–I; Figure S4D–G, Supporting Information). This result suggests a link between curcumin treatment and the mRNA levels of Parp14 and Nlrc3.

### 2.7 Parp14 is a Crucial Mediator in the Pathway by Which Curcumin Modifies Microglial Response

Our phosphoproteomics, MeRIP, and RNA‐seq data reveal that curcumin influences many intracellular proteins and pathways (Figures 3, 5). Among the various targets identified, we have focused specifically on Parp14 for further study. This decision is informed by existing research showing that Parp14 plays a critical role in mediating microglial activation.^[^ ^22^, ^23^ ^]^ This approach allows us to illustrate curcumin's regulatory impact using Parp14 as a representative example within a broader context.

**Figure 6:**
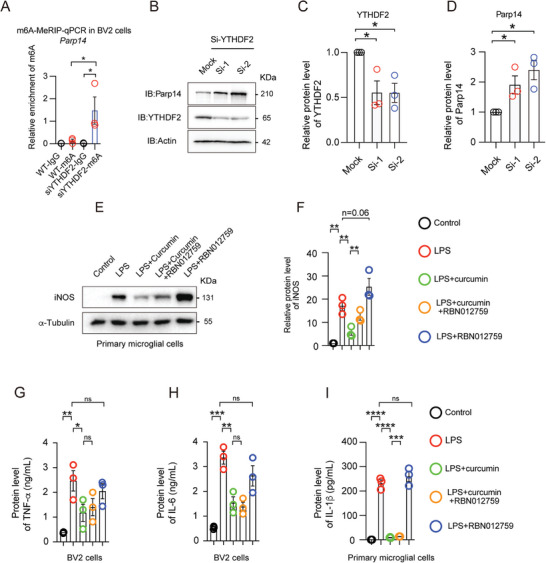
*Parp14* as a regulatory effector of YTHDF2. A) m6A modification enrichment in *Parp14* mRNA in BV2 cells was measured by MeRIP‐qPCR B) Western blotting of Parp14 in BV2 cells transfected with control siRNA or two individual YTHDF2 siRNAs. C,D) Protein quantification of Parp14 and YTHDF2 after knockdown of YTHDF2. Data are the mean ± SEM. *n =* 3 biologically independent experiments. *p < 0.05 by Student's *t*‐test. E) Western blot analyses of iNOS expression in primary microglia exposed to different combinations of LPS (250 ng mL^−1^), curcumin (10 µm), and RBN012759 (1 µm), or vehicle (control) in serum‐free medium for 16 h. F) iNOS expression levels under various conditions are shown. Data are mean ± SEM., *n =* 3 biologically independent experiments by Student's *t*‐test. G–I) Enzyme‐linked immunosorbent assay (ELISA) and a Mouse Proinflammatory V‐Plex Tissue Culture Kit analyses of TNF‐α, IL‐6 expression in BV2 and primary microglia cells treated with LPS (250 ng mL^−1^) alone or in combination with curcumin (10 µm) and/or RBN012759 (1 µm), or vehicle (control) for 16 h in serum‐free medium. Data are mean ± SEM., *n =* 3 biologically independent experiments by Student's *t*‐test.

First, to confirm whether YTHDF2 also regulates the Parp14 in microglial cells, we used BV2 cells to knock down YTHDF2 and studied the mRNA m6A modification level using MeRIP‐qPCR while measuring the protein level. Our results showed that the reduction of YTHDF2 significantly increased both the mRNA m6A modification and protein levels of Parp14 (**Figure** **6**A–D). These data suggest that the mRNA of Parp14 is a regulatory target of YTHDF2 in microglial cells.

To investigate the regulation of downstream factors YTHDF2 and Parp14 by PTPRZ1, we transfected a PTPRZ1‐Flag plasmid into 293T cells. Our results showed that overexpressed PTPRZ1 led to a decrease in YTHDF2 expression and an increase in Parp14 expression, suggesting a regulatory relationship consistent with our hypothesis (Figure S6A, Supporting Information).

Next, to evaluate the role of Parp14 protein in curcumin‐regulated microglial activation, we employed a model where primary microglia or BV2 cells were stimulated with LPS, LPS with curcumin, RBN012759 (a Parp14 inhibitor),^[^ ^24^ ^]^ or a combination of both (Figure 6E, and Figure S6B, Supporting Information). Our analysis focused on the expression of pro‐inflammatory factors such as inducible nitric oxide sythase (iNOS), TNF‐α, IL‐6, and IL‐1β, etc. We observed that curcumin significantly reduced the secretion of these inflammatory factors induced by LPS (Figure 6E–I, Figure S6B and S7A–C, Supporting Information). Interestingly, this inhibitory effect was substantially diminished when the Parp14 inhibitor was co‐administered, underscoring the critical role of Parp14 in mediating curcumin's anti‐inflammatory effects on microglia (Figure 6F,I; Figures S6B and S7B,C, Supporting Information). However, the administration of curcumin and RBN012759 did not affect the regulation of interferon gamma (IFN‐γ), interleukin12p70 (IL‐12p70), and interleukin 2 (IL‐2), suggesting selective regulation by curcumin (Figure S7D–F, Supporting Information).

Furthermore, the differential effects between the combination of curcumin with RBN012759, and the application of the RBN012759 alone, suggest that while Parp14 is a significant downstream modulator in curcumin's action, curcumin likely operates through additional pathways to regulate microglial activity. This complexity indicates that curcumin interacts with multiple targets, with Parp14 being a pivotal but not exclusive player. The overlapping and distinct pathways influenced by curcumin need further investigation to fully elucidate its broad regulatory mechanisms in microglial cells.

To further clarify the specific role of Parp14 in curcumin‐regulated microglial activation and to minimize the potential multi‐target effects of inhibitors, we knocked down Parp14 in BV2 cells before conducting LPS induction experiments (Figure S6C, Supporting Information). The results indicated that curcumin administration reduced the LPS‐induced increase in iNOS expression. Interestingly, this inhibitory effect was absent following the knockdown of Parp14. (Figure S6D–F, Supporting Information). These findings underscore Parp14 as a critical downstream mediator in the pathway through which curcumin modulates microglial response.

### 2.8 Curcumin May Influence Brain Microenvironment Homeostasis

**Figure 7:**
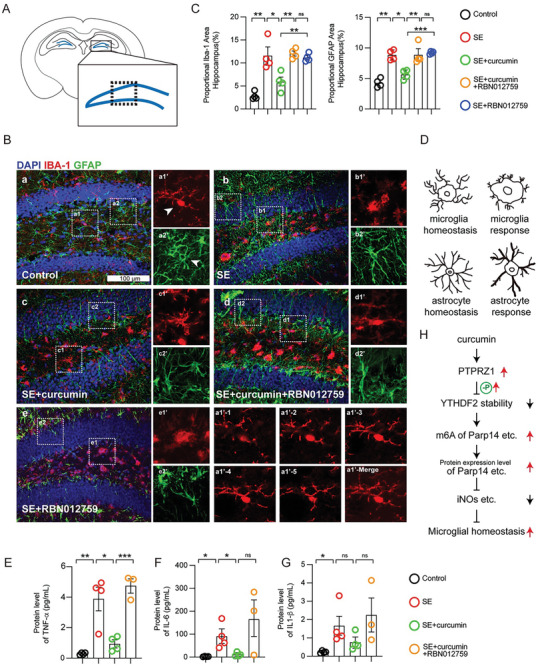
Parp14 is a vital downstream target of curcumin that maintains microglial homeostasis. A) Schematic diagram of the coronal section of mouse hippocampus. B) Iba‐1 and GFAP immunoreactivity was determined 72 h after intraperitoneal injection of saline or pilocarpine (290 mg kg^−1^) with or without curcumin (150 mg kg^−1^) and gavaged RBN012759 (100 mg kg^−1^). Representative images of labeling for IBA‐1 and GFAP (DG of the hippocampus) are shown in the five experimental groups. White arrowheads indicate a representative cell in the enlarged insert, scale bar 100 µm. a1’−1 to a1’−5 represent a microglia stack of confocal images (6 µm apart) from a1’ in the control group. C) Proportional area of IBA‐1 and GFAP in the DG of the hippocampus. Data are mean ± SEM., *n =* 4, *p < 0.05, **p < 0.01, ***p < 0.001, by Student's *t*‐test. D) Schematic diagram of microglia and astrocytes in homeostasis and activation. E–G) A Mouse Proinflammatory V‐Plex Tissue Culture Kit analyses of TNF‐α, IL‐6, IL‐1β expression in hippocampus and cortex from different groups. Data are mean ± SEM., *n =* 4 or *n =* 3, *p < 0.05, **p < 0.01, ***p < 0.001 by Student's *t*‐test. H) Flow chart of the research.

Under stress, microglia and astrocytes typically exhibit changes such as de‐ramification and astrogliosis (**Figure** **7**D). These changes are integral to maintaining brain microenvironment homeostasis and supporting central nervous system functions.^[^ ^25^ ^]^ Microglial morphological activation, including shortening and thickening of their processes concomitant with an enlargement of their somata, are consistent with a de‐ramified morphological profile.^[^ ^3^, ^25^ ^]^

To investigate the modulatory effect of curcumin on microglial immune reactivity through Parp14, we categorized mice into five groups: control, SE untreated, SE treated with curcumin, SE with RBN012759, and SE with both curcumin and RBN012759, and examined the effects of curcumin in epileptic mice, focusing on the dentate gyrus (DG) region of the hippocampus (Figure 7A,B).

Immunofluorescence staining of microglia and astrocytes indicated that the relative area of IBA1‐ and GFAP‐positive cells increased in the hippocampal region of SE mice treated with RBN012759 alone compared to SE mice treated with curcumin. However, this increase was not significantly different from the group receiving both RBN012759 and curcumin (p > 0.05). These results suggest that Parp14 plays a crucial role in the pathway through which curcumin modulates microglial activation. (Figure 7B,C). The same trend also applies to astrocytes and cytokines such as TNF‐α (Figure 7E). Whereas the addition of RBN02759 did not significantly reverse IL‐6, IL‐1β, and growth regulated alpha (KC/GRO) expression (Figure 7F,G and Figure S7G, Supporting Information), which was different from the primary microglia results (Figure 6I), this is possible because these cytokines are regulated by other molecular mechanisms.

These results demonstrate Parp14 as a critical but not solitary mediator in the pathway through which curcumin modulates microglial response. It is essential to recognize that while Parp14 is a significant modulator of curcumin's effects, curcumin interacts with multiple targets. This complexity necessitates further investigation to fully elucidate curcumin's broad regulatory mechanisms in microglial cells.

## 3 Discussion

In this study, we have identified that curcumin modulates the function of microglia through a direct interaction with PTPRZ1, which subsequently influences mechanisms of protein phosphorylation and m6A RNA modification. This interaction aids in regulating inflammatory responses, thereby expanding our understanding of curcumin's anti‐inflammatory mechanisms and offering a novel molecular basis for its effects under neuroinflammatory conditions.

PTPRZ1 plays a pivotal role in regulating cell signaling pathways that are crucial for various cellular functions, including cell proliferation, adhesion, and migration.^[^ ^26^ ^]^ Notably, within the nervous system, the activity of PTPRZ1 is essential for regulating neuronal responses and microglial functionality.^[^ ^16^ ^]^ Our study revealed that curcumin treatment and PTPRZ1 overexpression both attenuated LPS‐induced IL‐6 release in microglia (Figure 2E). However, their effects on TNF‐α differed curcumin significantly suppressed TNF‐α levels, while PTPRZ1 overexpression caused only a minor decrease that was not statistically significant (Figure 2F). This suggests that curcumin's anti‐inflammatory effects involve mechanisms beyond PTPRZ1 activation, particularly in the regulation of TNF‐α. Additionally, further optimization of experimental conditions allowed us to better evaluate the extracellular interaction between Flag‐PTPRZ1 and HA‐PTN in response to curcumin treatment (Figure 2A–C). These results confirm curcumin's ability to modulate the PTN‐PTPRZ1 interaction in a dose‐dependent manner, but remain limited to in vitro conditions, reflecting only extracellular interactions.

To further elucidate PTPRZ1's role, we examined the phosphorylation of its downstream targets, such as GIT1 (Figure 4A) and p190 RhoGAP (Figure S3C, Supporting Information), both of which are substrates regulated by PTPRZ1.^[^ ^27^ ^]^ These analyses confirmed that curcumin modulates PTPRZ1 activity, as indicated by altered phosphorylation states of these substrates. Importantly, the changes in GIT1 phosphorylation, which is critical for cytoskeletal dynamics and cellular responses,^[^ ^28^ ^]^ align with the observed effects of curcumin on microglial function. These findings suggest that while PTPRZ1 plays a role in curcumin's actions, additional pathways are likely involved.

Furthermore, the regulatory effects of curcumin on microglial activity are mediated through alterations in the m6A RNA modification pathway (Figure 5B–E). m6A modification plays a significant role in controlling RNA stability and translation, which is vital for cellular responses to environmental stimuli.^[^ ^29^ ^]^ YTHDF2, a critical protein that recognizes m6A modification sites, was observed to have altered phosphorylation states under the influence of curcumin (Figure 4A–G). This alteration potentially affects YTHDF2's ability to bind m6A‐modified RNA (Figure 4J), thereby modulating the expression of genes associated with inflammation (Figure 5C–F). While we highlighted Parp14 as a key example of curcumin's regulation within microglial cells, it is essential to emphasize that Parp14 is just one of the many targets through which curcumin exerts its regulatory effects.

Our study's insights into curcumin's interaction with PTPRZ1 and its impact on m6A RNA modification pathways not only enrich the existing knowledge of its pharmacological actions but also highlight its ability to exert broad regulatory effects at the molecular level. This could translate into novel therapeutic applications for curcumin in treating neuroinflammatory diseases.

Despite these promising findings, several limitations might affect the generalizability and interpretation of our results. The reliance on in vitro cell models and mouse models does not fully capture the complexity and heterogeneity of human diseases. The behavior of microglia in vitro might not accurately reflect their activity in more complex in vivo environments, potentially limiting the clinical relevance of our findings. Moreover, the concentrations of curcumin used in our in vitro experiments were considerably higher than those achievable in vivo, where peak plasma concentrations following oral administration range between 0.41 and 1.75 µm.^[^ ^30^ ^]^ This discrepancy underscores the need for novel curcumin formulations with improved bioavailability to achieve therapeutic effects against neuroinflammation.

Moving forward, it is important to further explore the potential of curcumin in treating neuroinflammatory diseases. High‐resolution structural biology techniques should be employed to provide a detailed view of the curcumin‐PTPRZ1 interaction and explore ways to modify the chemical structure of curcumin to enhance its stability and bioavailability. It is also important to expand research to include other models of neuroinflammation to help assess the broader applicability of curcumin. Furthermore, considering the potential synergistic effects of curcumin with existing drugs could open new avenues for combination therapies and enhance overall treatment strategies for neuroinflammatory conditions. By addressing these points, future research can lead to the development of new curcumin‐based drugs with higher bioavailability and enhanced therapeutic effects, tailored specifically to manage and treat neuroinflammatory disorders more effectively.

## 4 Experimental Section

### Antibodies and Reagents

Anti‐GIT1 (Santa Cruz, sc‐365084, 1:1000), anti‐PTPRZ1 (Santa Cruz, sc‐33664, 1:500), anti‐PTPRZ1 (BD, 610179, 1:1000), anti‐YTHDF2 (Cell Signaling Technology, 71283, 1:1000), anti‐m6A(Active Motif, 91262, 1:1500), anti‐m6A (SYSY, 202003, 1:2000), anti‐iNOS (Abcam, ab15323, 1:1000), anti‐Phosphotyrosine (4G10) (Milipore, 05–321X, 1:1000), anti‐IBA1 (WAKO, 019–19741, 1:1500), anti‐IBA1 (asis Biofarm, OB‐PGP049, 1:500), anti‐GFAP (Abcam, ab4674, 1:1000), anti‐Flag (Sigma Aldrich, F3165), anti‐HA (MBL, M132‐3, 1:3000), anti‐α‐tubulin (Sigma, T9026, 1:10000), anti‐β‐actin (Sigma, A5441, 1:8000), anti‐Parp14 (Santa Cruz, sc‐377150, 1:1000).

Curcumin (Solarbio, C7090), Recombinant Human Pleiotrophin (RayBio, 230–00618), RBN012759 (AIwei, 291347480), NAZ2329 (MedChemExpress, HY‐103693), Pilocarpine (Cayman, 14487), Scopolamine (Macklin, 6106‐46‐3), LPS (Sigma, 0111: B4 serotype, 14391), Phos‐tag agarose (WAKO, 308–93563), DYKDDDDK‐Nanoab‐Agarose (Lablead, FNA‐2‐40), Recombinant RNase Inhibitor (TaKaRa, 2313A), Recombinant Mouse beta‐NGF (Solarbio, P00114), Murine FGF‐basic (PEPROTECH, 450‐33‐10), Papain (Macklin, P6321), Dynabeads mRNA (Invitrogen, 61006), A Mouse Proinflammatory V‐Plex Tissue Culture Kit (Meso Scale Discovery, K15048D‐X), TNF‐α (Neobioscience, EMC102a.96), and IL‐6 (Neobioscience, EMC004.96).

### Animals

Male ICR mice weighing 30 ± 2 g were from Beijing Vital River Laboratory Animal Technology Co., Ltd. All mice were housed according to the Laboratory Animal Center, Institute of Genetics and Developmental Biology, Chinese Academy of Sciences guidelines. Mice were maintained in specific pathogen‐free conditions in the animal facility and housed on a 12‐h light/12‐h dark cycle with ad libitum access to food and water.

The pilocarpine‐induced SE model was developed by Turskis's group.^[^ ^31^ ^]^ In these experiments, pilocarpine (290 mg kg^−1^, i.p.) was injected intraperitoneal, and 30 min before that, scopolamine (1.5 mg kg^−1^) was injected into adult male ICR mice. After pilocarpine injection, the mice that developed status epilepticus (SE) was chosen that lasted up to 90 min, then injected diazepam (10 mg kg^−1^), which was given to terminate the seizures. Mice with seizures greater than or equal to grade IV were used in the study, which was treated with or without intraperitoneal injection curcumin (150 mg kg^−1^) and gavaged RBN012759 (100 mg kg^−1^).

The progressive evolution of seizures was similar to that classified by Racine in the kindling model.^[^ ^32^ ^]^ Mice were sacrificed 72 h after SE for research.

### Affinity Chromatography and Mass Spectrometry—Immobilization of Curcumin on Epoxy‐Activated Sepharose 6B

The establishment of stationary phases for coupling curcumin to epoxy‐activated Sepharose 6B (EAS6B) was done according to previous methods.^[^ ^33^ ^]^ 1 g of EAS6B was washed in 50 ml of cold distilled water for five cycles, with each cycle including centrifugation at 1500 g for 5 min, and then approximately 3.5 mL of swollen EAS6B was collected in 20% ethanol and stored at 4 °C for backup. Curcumin was dissolved in a coupling buffer containing 100 mm Na_2_CO_3_, 10 mm NaOH, and 50% dimethylformamide to a final concentration of 20 mm. Two volumes of the Curcumin coupling buffer were mixed with one volume of swollen beads and shaken overnight at 30 °C in the dark. The next day, the curcumin‐sepharose suspension was washed five times with coupling buffer and the remaining non‐specific binding sites were blocked by incubation with 1 m glycine overnight at 30 °C. Control beads were prepared by incubating the epoxy‐activated Sepharose 6B with 1 m glycine. The control beads and curcumin‐coupled beads were applied to the drip column. Subsequent washing steps were performed according to the manufacturer's instructions.

### Preparation of Mouse Brain Lysates

Mice were anesthetized perfused with saline on ice via the heart, and the brain was stripped from the skull and the brain stem was removed. The brain was flushed with PBS on ice, and a lysis buffer containing 50 mm HEPES (pH 7.45), 50 mm NaCl, 1 mm EDTA, 2 mm DTT, 1 mm PMSF, and 1%PIC was added at a mass‐to‐volume ratio of 1: 4. Tissue homogenization was accomplished with a tissue disperser and then a glass homogenizer. Samples were loaded into a pre‐chilled centrifuge and spun at 600 g for 20 min, and the supernatant was subjected to ultracentrifugation at 4 °C, 150 000 g for 60 min.

### Desalination and Decontextualization of Protein

Desalting buffer containing 20 mm HEPES (pH 7.45), 150 mm CH_3_COOK, 1 mm EDTA, 1 mm PMSF, 2 mM DTT, and 5 mm MgSO_4_, was diluted 1000 times for protein samples, and samples were then concentrated with 3 KD ultrafiltration tubes. 100 mm cellobiose, 0.1% Sarcosine, and 1% PIC were added to the desalted sample and then stored on ice. To prepare the decontextualization column, it was filled with 10 mL of Sepharose 6B, washed 5 times with one column volume of cold ultrapure water, and then replaced 3 times the column volume of desalting buffer to equilibrate the column. The desalted sample flowed through the decontextualization column to remove non‐specific binding sites. The column was washed with desalting buffer, and the flow‐through solution was collected, using the Bradford assay to check the protein concentrations. The flow‐through solution was concentrated with a 3 KD ultrafiltration tube to 800 uL and 100 mm cellobiose, 0.1% sarcosine, and 1% PIC was added, and the final protein concentration was determined by BCA. The final sample was divided into two parts and stored on ice until use.

### Protein Purification From Stationary Phases

The column was equilibrated with 5 times the column volume of the desalting buffer, and when the liquid was finished flowing, the final sample was added. The column was rinsed with 8 times the column volume desalting buffer and washing was stopped when Bradford detected no further protein in the flow‐thru. Protein was eluted by a gradient of 2 times the column volume of 0.3 m NaCl, 0.6 m NaCl, 2 m NaCl, and 2 m NaHS. 10% SDS was added to make a final concentration of 1%, and the sample was heated at 95 °C for 5 min. Silver staining was used to check the sample, and then samples were analyzed by mass spectrometric detection.

### Immunofluorescence

Mice were anesthetized and subjected to transcardial perfusion with PBS followed by 4% paraformaldehyde (PFA). Brains were postfixed in 4% PFA for 24 hours and submerged in 30% sucrose in PBS for 48 hours. Preserved brains embedded in OCT media were stored at −80 °C and sectioned (30 µm) using a LEICA CM 1950 cryostat. Brain sections were identified by the stereotaxic mouse brain atlas.^[^ ^34^ ^]^ The sections were washed with PBS thrice at room temperature and then incubated with the blocking solution (0.1% Triton X‐100 in PBS with 3.5% goat serum) for an hour before incubating them with primary antibodies overnight at 4 °C with rocking. After washing with PBST, the sections were incubated with secondary antibodies for 1.5 hours and then washed thrice with PBST for 5 min each. Finally, the sections were mounted using a cover glass.

Fluorescent images were examined using an Olympus FV3000 microscope for the assessment of microglia and astrocyte phenotypic changes in the hippocampal dentate gyrus (DG) region via Iba‐1 and GFAP staining, respectively. For each mouse, 6–8 representative images in the dentate gyrus regions were captured. Fiji software was utilized to establish the positive threshold, which included cell bodies and processes while excluding background staining. The average area percentage of positive threshold staining across all representative images was reported.

### Cell Culture

The immortalized mouse glial cell line BV‐2 was a generous gift from Dr. Weixiang Guo. The HEK293T cell lines were from our laboratory. These cell lines were cultured in Dulbecco's modified Eagle's medium (DMEM) supplemented with 10% fetal bovine serum (FBS) and 1% P/S at 37 °C in 5% CO2.

### Plasmid Construction

The full length of the pCAH‐PTPRZ1‐flag plasmid (human accession number: NM_002851.3), was chemically synthesized and cloned into vector pCAH‐flag using the EcoRI and BamHI sites. The FLAG was inserted before the stop codon of the putative open reading frame (ORF). The homologous recombination strategy was adopted.

Forward primer: cgtacggttcggatcgatatcatgcgaatcctaaagcgtttcc,

Reverse primer: gtccttgtagtcgacgcggccgcaaactaaagactctaagctctcagctatatt.

pCDNA3.1‐PTN‐TurboID‐HA plasmid (human accession number: NM_001321386.2) was cloned into the vector pCDNA3.1‐HA (‐) using the NheI and KpnI sites fusing insert sequence TurboID behind the PTN coding sequence.

Forward primer: atagggagacccaagctgatgcaggctcaacagtaccagcagcag,

Reverse primer: agtattgtctttgctagcatccagcatcttctcctgtttcttgc.

pCMV‐YTHDF2‐WT, pCMV‐YTHDF2‐S2A, and pCMV‐YTHDF2‐S2D plasmids (mouse accession number: NM_145393.4), were cloned into the vector pCMV‐Tag2B using the BamHI and EcoRI sites. The second serine (Ser, S) of YTHDF2 was mutated to alanine (Ala, A) for non‐phosphorylated activity or aspartic acid (Asp, D) for phosphorylated activity.

Forward primer: aagagcccgggcggatccatgtcggccagcagcctc,

Reverse primer: aagcttgatatcgaattcctatttcccacgaccttg.

### RNA Interference and Plasmid Transfection

For RNA interference in BV2 Microglial Cells via Lipofectamine 2000, BV2 cells were cultured to 50–60% confluency before transfection. On the day of transfection, the manufacturer's instructions were followed. Nucleotide sequences of siYTHDF2‐1: 5′‐ GCACAGAGCAUGGUAACAATT −3′; siYTHDF2‐2: GGACGUUCCCAAUAGCCAATT; siPARP14: 5′‐ CAGCAAUAGGAACGGGAAATT; siControl: 5′‐

UUCUCCGAACGUGUCACGUTT‐3′.

For DNA overexpression, HEK293T cells or BV2 cells were transfected using Lipofectamine 2000 reagent according to the manufacturer's instructions when the cells had reached 70%−80% confluency.

### Cytokines Assay

To examine whether PTPRZ1 regulates microglial response, an enzyme‐linked immunosorbent assay (ELISA) was performed. Briefly, BV2 cells overexpressed PCAH‐PTPRZ1‐Flag plasmid and vector PCAH‐Flag plasmid for 48 h. The cells were treated with LPS (250 ng mL^−1^), LPS (250 ng mL^−1^) + curcumin (10 µm), LPS (250 ng mL^−1^) + NAZ2329 (25 µm), or vehicle (PBS with 1‰ DMSO) in serum‐free medium for 16 h. The levels of TNF‐α (EMC102a.96) and IL‐6 (EMC004.96) in the collected culture medium were analyzed using a commercial ELISA kit (Neobioscience, Shenzhen, China) according to the manufacturer's instructions.

To explore the influence of Parp14 on microglia‐mediated inflammation response, the BV2 cells were subjected to serum starvation for 24 h in 0.1% FBS, then treated with 1 µm RBN012759, a Parp14 inhibitor (CAS:2360851‐29‐0, 291347480), or with DMSO as the vehicle (0.1% final concentration in each well) 6 h before stimulation with 250 ng mL^−1^ LPS for an additional 16 h. The cytokines TNF‐α and IL‐6 were measured using an ELISA kit as described above.

To detect proinflammatory cytokine secretion in primary microglia, culture medium was collected and centrifuged in the LPS (250 ng mL^−1^), LPS (250 ng mL^−1^) + curcumin (10 µm), LPS (250 ng mL^−1^) + curcumin (10 µm) + RBN012759 (1 µm), LPS (250 ng mL^−1^) + RBN012759 (1 µm), and vehicle group. A Mouse Proinflammatory V‐Plex Tissue Culture Kit (Meso Scale Discovery, Rockville, MD) was used to measure IFN‐γ, IL‐1β, IL‐2, IL‐4, IL‐5, IL‐6, IL‐10, IL‐12p70, KC/GRO, TNF‐α concentrations according to the manufacturer's instructions. All experiments were repeated at least three times in biological replicates.

### RNA m6A Dot Blot Assays

To detect the effect of curcumin phosphorylation sites on the level of m6A (N6‐methyladenosine) RNA modifications, the total m6A levels in RNA were measured by dot blot assays, according to previous methods.^[^ ^35^ ^]^ Total RNA extracted from BV2 cells or mice hippocampus was adjusted to 50 ng/µL with 36 µL RNase‐free water. After removing the RNA secondary structure, samples were chilled on ice and then loaded onto an N^+^ membrane (GE Health) optimized for nucleic acid transfer. It was cross‐linked by ultraviolet radiation (1200 microjoules, 30 sec), stained by methylene blue (Sigma‐Aldrich), and then incubated with an m6A antibody.

### MeRIP‐seq and Data Analysis

To investigate the effect of curcumin on the RNA m6A modification fragments, MeRIP‐seq was performed by SeqHealth Technology Co., Ltd. (Wuhan, China) following previous methods.^[^ ^36^ ^]^ Briefly, total RNA from the hippocampus of the mouse was extracted with TRIzol (Invitrogen, cat. NO. 15596026). Using a NanoDrop ND‐1000 to assess RNA quality through 1.5% agarose gel electrophoresis to confirm RNA integrity, finally qualified RNAs by QubitTM RNA Broad Range Assay kit with the Qubit3.0.  50 µg of total RNA were enriched for polyadenylated RNA using VAHTS mRNA Capture Beads (VAHTS, cat. NO. N401‐01/02). mRNA was incubated at 95°C for 5min‐10 min until the fragments were mainly distributed in 100–200 nt, of which 10% was retained as input sample, while the rest was subjected to a specific anti‐m6A antibody (Synaptic Systems, 202203) for m6A immunoprecipitation (IP). A library was constructed using the KC‐DigitalTM Stranded mRNA Library Prep Kit for Illumina (Cat. NO. DR08502, Wuhan Seqhealth Co., Ltd. China), following the manufacturer's instructions, and finally sequenced on DNBSEQ‐T7 sequencer (MGI Tech Co., Ltd. China). Low‐quality reads were eliminated with Trimmomatic (version 0.36), and the high‐quality clean reads were aligned to the reference genome (UCSC GRCm38) with STAR software (version 2.5.3a). The peak calling was done with the exomePeak software (Version 3.8). The m6A peaks were annotated with bedtools (Version 2.25.0), and a peak distribution analysis was performed with deepTools (Version 2.4.1). Sequence motifs enriched in m6A peak regions were identified using Homer (version 4.10) with a corrected p‐value cutoff of 0.05 as the threshold for statistical significance.

### MeRIP‐qPCR

Mice brain or BV2 cells were used for total RNA extraction. Intact poly‐A RNA was purified from total RNA and subjected to fragmentation at 94 °C for 30 s, the mRNA was collected by ethanol precipitation. Then, the mRNA was incubated with m6A antibody, and RNase inhibitor in IPP^[^ ^41^, ^42^ ^]^ buffer (10 mm Tris‐HCl, PH = 7.4, 150 mm NaCl, 0.1% NP‐40) and complexes were captured using Sepharose Protein A (GE Healthcare, 17‐5280‐01) at 4 °C for 3 h with rotation. The Sepharose beads captured m6A mRNA complexes were washed twice with IPP buffer, and then, mRNA was extracted by TRIzol. Precipitated mRNA was reverse‐transcribed and quantified by qPCR for m6A enrichment.

### RNA‐seq and Data Analysis

High‐throughput sequencing of RNA from the hippocampus of the mouse was performed by the Beijing Genomics Institute (BGI, China). Briefly, total RNA from the hippocampus of the mouse was extracted with TRIzol (Invitrogen, 15596026). following the manufacturer's instructions. RNA libraries were constructed by using a Hieff NGS Ultima Dual‐mode RNA Library Prep Kit for MGI according to the manufacturer's instructions. Library sequencing was performed on an Illumina NovaSeq 6000 instrument with 150 bp paired‐end reads. Paired‐end reads were harvested from the Illumina NovaSeq 6000 sequencer and were quality‐controlled by Q30. After 3′ adaptor trimming and removal of low‐quality reads with SOAPnuke (v1.5.2), the high‐quality clean reads were aligned to the reference genome (UCSC GRCm39) with HISAT2 software (v2.0.4). Data analyzed and visualized with Dr.Tom (https://biosys.bgi.com/#/report/login)

### LC‐MS/MS Analysis and Data Analysis

Proteins were digested using the filter‐aided sample preparation (FASP) method with slight modifications.^[^ ^37^ ^]^ After reduced and alkylated with DTT and iodoacetamide, the lysates were transferred to the Microcon YM‐30 centrifugal filter units (EMD Millipore Corporation, Billerica, MA) to replace the buffer with 200 ul UA (8 m Urea, 100 mm Tris‐Cl pH8.5) twice, and then replace with 0.1 m triethylammonium bicarbonate (TEAB, Sigma‐Aldrich, Saint Louis, MO). After the buffer was replaced, the proteins were digested with sequencing grade trypsin (1:50 (w: w)) at 37°C overnight. The resultant tryptic peptides were desalted by StageTips,^[^ ^38^ ^]^ and completely dried with a SpeedVac concentrator, and stored at −20 °C for later analysis. For MS analyses, peptides were resuspended in 0.1% FA (Formic acid) and analyzed by LTQ Orbitrap Elite mass spectrometer (Thermo Fisher Scientific) coupled online to an Easy‐nLC 1000 (Thermo Fisher Scientific) in the data‐dependent mode. The peptides were separated by reverse phase LC with a 150 µm (ID) ×250 mm (length) analytical column packed with C18 particles of 1.9 µm diameter. The mobile phases for the LC contain buffer A (0.1% FA) and buffer B (100% ACN, 0.1% FA), and a non‐linear gradient of buffer B from 3%−30% for 90 min was used for the separation. Precursor ions were measured in the Orbitrap analyzer at 240 000 resolution (at 400 m/z) and a target value of 106 ions. The twenty most intense ions from each MS scan were isolated, fragmented, and measured in the linear ion trap. The CID normalized collision energy was set to 35.

### Phosphoproteomic Analysis and Data Analysis

The cells were lysed in a buffer containing 4% SDC and 100 mm Tris‐HCl (pH 8.5). Proteins were reduced and alkylated by Tris (2‐carboxyethyl) phosphine (TCEP) and 2‐Chloroacetamide (CAA), and digested with sequencing grade trypsin (1:50 w/w) at 37 °C overnight. After digestion, the phosphopeptides were enriched using titanium dioxide beads (TiO2; GL Sciences, 5010–21315) following EasyPhos workflow.^[^ ^39^ ^]^ For data analyses, the resuspended peptides were analyzed by Orbitrap Fusion Lumos Tribrid mass spectrometer (Thermo Fisher Scientific) coupled online to an Easy‐nLC 1000 (Thermo Fisher Scientific) in the data‐dependent mode. The peptides were separated by reverse phase LC with a 150 µm (ID) ×250 mm (length) analytical column packed with C18 particles of 1.9 µm diameter. The mobile phases for the LC contain buffer A (100% H_2_O, 0.1% FA) and buffer B (100% ACN, 0.1% FA), and a 110‐min non‐linear gradient was used for the separation. All MS measurements were performed in the positive ion mode. Precursor ions were measured in the Orbitrap analyzer at 240 000 resolution (at 200 m/z) and a target value of 10^6^ ions. The twenty most intense ions from each MS scan were isolated and fragmented by high‐energy collisional dissociation and measured in the linear ion trap. The database search was performed for all raw MS files using the software MaxQuant (version 1.6.3.4). The Mus musculus proteome sequence database from UniProt was applied to search the data. Serine, threonine, and tyrosine phosphorylation, protein N‐terminal acetylation, and methionine oxidation were included in the search as the variable modifications. Cysteine carbamidomethylation was set as stable modifications.

### Quantitative PCR with Reverse Transcription

Total RNA was extracted from BV2 cells and mice using TRIzol reagent (YTHX biotech, YD003) or RNA kit (TRAN, ER101‐01). RNA was isolated according to the manufacturer's instructions. Reverse transcription was performed according to the manufacturer's instructions for the HiScript III 1st Strand cDNA Synthesis Kit (+gDNA wiper) (Vazyme, R312‐01/02). Gene expression levels were determined by real‐time PCR using Taq Pro Universal SYBR qPCR Master Mix (Vazyme, Q712) and CFX384 Real‐Time System (Bio‐Rad). The sequences of primers used for the detection of mRNA transcripts were listed below:

**Table:** 

Actin‐F:	AAGGCCAACCGTGAAAAGAT
Actin‐R:	GTGGTACGACCAGAGGCATAC
GAPDH‐F:	AGGTCGGTGTGAACGGATTTG
GAPDH‐R:	TGTAGACCATGTAGTTGAGGTCA
IL‐1β‐F:	GCCCATCCTCTGTGACTCAT
IL‐1β‐R:	TTGTCGTTGCTTGGTTCTCC
NLRC3‐F:	GTGGACCGGATGACTGAGAT
NLRC3‐R:	TACAAGTGACCCAGAGCCAG
Parp14‐F	CTTTCCCACACAGCTTTCCC
Parp14‐R	CACAATGGCATGGGTCGTAG

Data were analyzed using the Sequence Detection Software according to the ΔCt method. All results were normalized to the corresponding values for Actin or Gapdh quantified in parallel amplification reactions. In each experiment, at least three biological samples were analyzed.

### Western Blotting and Quantification

The sample was separated by SDS‐PAGE, followed by semidry electroblotting onto a polyvinylidene difluoride membrane (PVDF). After blocking with 5% nonfat dry milk with 0.1% Tween 20 in TBS, membranes were incubated overnight with the respective primary antibodies. After incubation with appropriate secondary antibodies conjugated with horseradish peroxidase at room temperature for 1.5 h, the immunoreactive proteins were visualized using the ECL method according to the manufacturer's instructions.

Protein levels were quantified using Fiji software (NIH, Bethesda, USA). The bands corresponding to the respective proteins were manually delineated, and the signal intensity was measured. The total gray signal in each band was quantitated and normalized to the intensity of α‐Tubulin or Actin. The intensities were also measured using Fiji. The graphs display the intensities as values relative to each experiment's maximum intensity (100%).

### Cycloheximide (CHX) Chase Analysis

To study the effect of phosphorylation level at the YTHDF2‐S2 (WT) site on its stability, 293T cells overexpressed pCMV‐Tag2B‐fused plasmids for 24 h. YTHDF2‐WT, YTHDF2‐S2A, and YTHDF2‐S2D overexpressing cells cultured without FBS were then incubated with 100 µg mL^−1^ of cycloheximide at various times. Cell lysates were prepared using RIPA buffer and subjected to western blots.

### Co‐immunoprecipitation (co‐IP)

To investigate the effect of curcumin on PTN‐PTPRZ1 interaction. 293T cells were transfected with pCMV‐Tag2B or pCMV‐Tag2B‐fused plasmids, and pCDNA3.1‐PTN‐TurboID‐HA plasmid for 18 h. Then cells were lysed for 30 min at 4 °C in a buffer containing 50 mm Tris‐HCl, pH 8.0; 150 mm NaCl; 1 mm EDTA; 1% NP‐40, phosphatase Inhibitor Cocktail (Roche, 04906837001), and protease inhibitors (Mei5bio, MF182‐plus). The lysates were centrifuged for 15 min at 12,000 rpm, and the supernatants were collected and incubated with 10 µL of anti‐FLAG M2 affinity gel (GE Healthcare) at 4 °C. After incubating for 1.5 h, the lysates were centrifuged for 5 min at 3,000 rpm, 4 °C. After which, the beads were collected and washed three times in wash buffer containing 20 mm Tris‐HCl, pH 8.0; 150 mm NaCl; 1 mm EDTA; 0.1% NP‐40. The beads were evenly divided into 4 parts in PBS, and different concentrations of curcumin were added and incubated at 37 °C for 30 min, and then beads were washed three times in the wash buffer. The collected protein complexes were boiled in 1 × sample buffer containing 50 mM Tris‐HCl (pH 6.8), 100 mm DTT, 2% (w/v) SDS, 0.1% (w/v) Bromoxylenol blue, and 10% (v/v) glycerol, and detected using western blotting.

### Immunoprecipitation (IP)

To explore the effect of curcumin on PTPRZ1 enzyme activity. GIT, the substrate of PTPRZ1, was selected to reflect the enzyme activity of PTPRZ1 through its phosphorylation. 293T stable and BV2 cells were lysed for 30 min at 4 °C in the buffer containing 50 mm Tris‐HCl, pH 8.0; 150 mm NaCl; 1 mm EDTA; 1% NP‐40, phosphatase Inhibitor Cocktail (Roche, 04906837001), and protease inhibitors (Mei5bio, MF182‐plus). The lysates were centrifuged for 15 min at 12,000 rpm, and the supernatants were collected and incubated with GIT1 antibody 5 ul for 1.5 h, then incubated with 8 µL Protein G Sepharose (GE Healthcare) for 1.5 h. The lysates were centrifuged for 5 min at 3,000 rpm, 4 °C. After this, the beads were collected and washed three times in the wash buffer, and the collected protein complexes were boiled in 1 × sample buffer and detected using western blotting.

### Phosphate Affinity Chromatography using Phos‐tag Agarose

To verify phosphorylation mass spectrometry data, BV2 cells were plated in 6 wells at a cell density of 5 × 10^5^ per well, and primary microglia were plated in 12 wells at a cell density of 5 × 10^5^ per well. After 12 h, when cells lie on the bottom, cells were subjected to serum starvation for 12 h, treated with PTN (100 nm), curcumin (20 µm), NAZ2329 (10 µm), or vehicle (control) in DMEM without FBS for 20 min. Cells were lysed for 30 min at 4 °C in a buffer containing 50 mm Tris‐HCl, pH 7.5; 150 mm NaCl; 1 mm EDTA; 1% NP‐40, 0.25% SOD, 1 mm PMSF, 1 mm Na_3_VO_4_, 1 mm NaF, phosphatase Inhibitor Cocktail, and protease inhibitors (Mei5bio, MF182‐plus). The lysates were centrifuged for 15 min at 12 000 rpm, the supernatants were collected, and protein concentration was determined using a BCA kit (Solarbio, PC0020) to ensure that the protein content was equal between groups. Then the manufacturer's instructions were followed.

### Biological Information Analysis

The Venn analyses were completed using an online tool (https://bioinfogp.cnb.csic.es/tools/venny/index.html). The cluster, scatter plots, and volcano plots analyses used other online tools (https://hiplot.com.cn/home/index.html). The QIAGEN Ingenuity Pathway Analysis (IPA) platform was used to evaluate the upstream and downstream relationship of phosphorylation mass spectrometry results. KEGG pathway and GO analyses were conducted using the Metascape database (http://metascape.org/). When p<0.01, the minimum number of annotated genes was 3 as the condition for screening.

### Primary Cultures of Microglia

Microglia were isolated from postnatal day 0 to day 1 mice according to a protocol^[^ ^40^ ^]^ with slight modifications. Briefly, all pups were decapitated with surgical scissors, and the skull was cut along with the medulla oblongata, the cerebellum, and olfactory bulbs were cut off and the meninges were carefully removed, the remaining brain tissue was transferred to a new chilled 6‐well plate containing 2 mL of pre‐chilled HBSS (Hanks’ balanced salt solution). Two brain tissues were placed in a 2 mL tube with 1 mL digestion buffer (containing 8 U/ mL papain and 125 U mL^−1^ DNase to HBSS) and the brain tissue was fully minced into small pieces (approximate 1 mm^2^) with the help of spring scissors. The tube was placed in the 5% CO2, 37 °C humidified incubator for 20 min, and was swirled every 10 min. The digestion was terminated by adding a 1 mL culture medium (DMEM with 10% FBS and 1% P/S) to each tube. To remove big clumps and cell debris, the cell suspension was carefully passed through a 70 µm cell strainer and the flow‐through was collected in a 50 mL collection tube and then transferred to a 15 mL tube. Cells were then centrifuged at 1000 rpm for 5 min at room temperature and resuspended in 4 mL culture medium in a T25, this was Day 0. The culture medium was changed on the next day (Day 1) to microglia medium (DMEM with 20% FBS, 1% P/S, 10 ng/µL FGF, 10 ng/µL NGF), and 3–4 days later, the culture medium was changed again and at about a week after that, the microglia were harvested by shaking the flasks on a laboratory shaker at 180 rpm for 30 min.

### Statistical Analyses

Statistical tests were performed using GraphPad Prism 9 software. The two‐tailed student's t‐test was used to compare the two groups. All conditions were statistically different from the control indicated (*p < 0.05, **p < 0.01, ***p < 0.001; N.S., not significant, p > 0.05). All experiments were performed with at least three biological replicates as was also described in each figure legend.

### Ethical Approval

All animal experiments were approved by the Animal Center of the Institute of Genetics and Developmental Biology (IGDB), Chinese Academy of Sciences, and by the Institutional Animal Care and Use Committee (IACUC) and were conducted in accordance with the IACUC guidelines at IGDB.

## Conflict of Interest

The authors declare no conflict of interest.

## Author Contributions

N.Z., Q.X, and W.M. designed all experiments, interpreted the results, and prepared the manuscript. N.Z. performed most of the experiments and data analysis. R.L. performed the ELISA experiment and data analysis. W.G. performed the Parp14 knocked‐down experiment and data analysis. X.H. and Y.W. contributed to the mass spectrometric analysis. Y.L., H.X., and X.J. provided advice.

## Supporting information



Supporting Information

Supporting Information

## Data Availability

The data that support the findings of this study are available from the corresponding author upon reasonable request.

## References

[1] L. L. Quan , A. Uyeda , R. Muramatsu , Inflamm Regen 2022, 42, 7. 35232486 10.1186/s41232-022-00193-y PMC8888026

[2] I. D. Vainchtein , A. V. Molofsky , Trends Neurosci. 2020, 43, 144. 32044129 10.1016/j.tins.2020.01.003 PMC7472912

[3] U. B. Eyo , M. Murugan , L. J. Wu , Glia 2017, 65, 5. 27189853 10.1002/glia.23006 PMC5116010

[4] W. Wu , Y. Li , Y. Wei , D. B. Bosco , M. Xie , M. G. Zhao , J. R. Richardson , L. J. Wu , Brain Behav Immun 2020, 89, 245. 32621847 10.1016/j.bbi.2020.06.028 PMC7572576

[5] a) A. Dhir , Phytother Res 2018, 32, 1865; 29917276 10.1002/ptr.6125

[6] a) J. K. Kleen , G. L. Holmes , Nat. Med. 2008, 14, 1309; 19057551 10.1038/nm1208-1309

[7] M. Karlstetter , E. Lippe , Y. Walczak , C. Moehle , A. Aslanidis , M. Mirza , T. Langmann , J Neuroinflammation 2011, 8, 125. 21958395 10.1186/1742-2094-8-125 PMC3192695

[8] a) G. Curia , D. Longo , G. Biagini , R. S. Jones , M. Avoli , J Neurosci Methods 2008, 172, 143; 18550176 10.1016/j.jneumeth.2008.04.019 PMC2518220

[9] N. Maeda , M. Noda , Development 1996, 122, 647. 8625816 10.1242/dev.122.2.647

[10] A. Henn , ALTEX 2009, 26, 83. 19565166 10.14573/altex.2009.2.83

[11] a) S. Zhang , F. Liang , B. Wang , Y. Le , H. Wang , Acta Histochem. 2014, 116, 415; 24157126 10.1016/j.acthis.2013.09.003

[12] M. Fukada , H. Kawachi , A. Fujikawa , M. Noda , Methods 2005, 35, 54. 15588986 10.1016/j.ymeth.2004.07.008

[13] K. Kuboyama , A. Fujikawa , R. Suzuki , N. Tanga , M. Noda , J. Biol. Chem. 2016, 291, 18117. 27445335 10.1074/jbc.M116.742536 PMC5000061

[14] F. Ghasemi , H. Bagheri , G. E. Barreto , M. I. Read , A. Sahebkar , Neurotox Res 2019, 36, 12. 30949950 10.1007/s12640-019-00030-0

[15] R. Orihuela , C. A. Mcpherson , G. J. Harry , Br. J. Pharmacol. 2016, 173, 649. 25800044 10.1111/bph.13139 PMC4742299

[16] R. Fernandez‐Calle , M. Galan‐Llario , E. Gramage , B. Zapateria , M. Vicente‐Rodriguez , J. M. Zapico , B. de Pascual‐Teresa , A. Ramos , M. P. Ramos‐Alvarez , M. Uribarri , M. Ferrer‐Alcon , G. Herradon , Sci. Rep. 2020, 10, 20259. 33219280 10.1038/s41598-020-76415-5 PMC7679445

[17] A. Fujikawa , H. Sugawara , N. Tanga , K. Ishii , K. Kuboyama , S. Uchiyama , R. Suzuki , M. Noda , J. Biol. Chem. 2019, 294, 14953. 31416834 10.1074/jbc.RA119.007878 PMC6791311

[18] a) S. J. Humphrey , D. E. James , M. Mann , Trends Endocrinol. Metab. 2015, 26, 676; 26498855 10.1016/j.tem.2015.09.013

[19] L. Gan , Y. Zhao , Y. Fu , Q. Chen , Biomed. Pharmacother. 2023, 160, 114343. 36758318 10.1016/j.biopha.2023.114343

[20] R. Fang , X. Chen , S. Zhang , H. Shi , Y. Ye , H. Shi , Z. Zou , P. Li , Q. Guo , L. Ma , C. He , S. Huang , Nat. Commun. 2021, 12, 177. 33420027 10.1038/s41467-020-20379-7 PMC7794382

[21] H. L. Sun , A. C. Zhu , Y. Gao , H. Terajima , Q. Fei , S. Liu , L. Zhang , Z. Zhang , B. T. Harada , Y. Y. He , M. B. Bissonnette , M. C. Hung , C. He , Mol. Cell 2020, 80, 633. 33217317 10.1016/j.molcel.2020.10.026 PMC7720844

[22] a) J. Xu , C. Gao , Y. He , X. Fang , D. Sun , Z. Peng , H. Xiao , M. Sun , P. Zhang , T. Zhou , X. Yang , Y. Yu , R. Li , X. Zou , H. Shu , Y. Qiu , X. Zhou , S. Yuan , S. Yao , Y. Shang , Mol. Ther. 2023, 31, 154; 36068919 10.1016/j.ymthe.2022.08.023 PMC9840117

[23] Y. Tang , J. Liu , Y. Wang , L. Yang , B. Han , Y. Zhang , Y. Bai , L. Shen , M. Li , T. Jiang , Q. Ye , X. Yu , R. Huang , Z. Zhang , Y. Xu , H. Yao , Autophagy 2021, 17, 2905. 33317392 10.1080/15548627.2020.1847799 PMC8525999

[24] L. B. Schenkel , J. R. Molina , K. K. Swinger , R. Abo , D. J. Blackwell , A. Z. Lu , A. E. Cheung , W. D. Church , K. Kunii , K. G. Kuplast‐Barr , C. R. Majer , E. Minissale , J. R. Mo , M. Niepel , C. Reik , Y. Ren , M. M. Vasbinder , T. J. Wigle , V. M. Richon , H. Keilhack , K. W. Kuntz , Cell Chem. Biol. 2021, 28, 1158. 33705687 10.1016/j.chembiol.2021.02.010

[25] D. M. Norden , P. J. Trojanowski , E. Villanueva , E. Navarro , J. P. Godbout , Glia 2016, 64, 300. 26470014 10.1002/glia.22930 PMC4707977

[26] N. K. Tonks , FEBS J. 2013, 280, 346. 23176256 10.1111/febs.12077 PMC3662559

[27] a) M. Fukada , M. Noda , Methods Mol Biol 2007, 365, 371; 17200575 10.1385/1-59745-267-X:371

[28] B. Huck , R. Kemkemer , M. Franz‐Wachtel , B. Macek , A. Hausser , M. A. Olayioye , J. Biol. Chem. 2012, 287, 34604. 22893698 10.1074/jbc.M112.374652 PMC3464566

[29] S. Zaccara , R. J. Ries , S. R. Jaffrey , Nat. Rev. Mol. Cell Biol. 2019, 20, 608. 31520073 10.1038/s41580-019-0168-5

[30] S. Prasad , A. K. Tyagi , B. B. Aggarwal , Cancer Res. Treat. 2014, 46, 2. 24520218 10.4143/crt.2014.46.1.2 PMC3918523

[31] a) L. Turski , C. Ikonomidou , W. A. Turski , Z. A. Bortolotto , E. A. Cavalheiro , Synapse 1989, 3, 154; 2648633 10.1002/syn.890030207

[32] R. J. Racine , Electroencephalogr Clin Neurophysiol 1972, 32, 281. 4110397 10.1016/0013-4694(72)90177-0

[33] L. Conboy , A. G. Foley , N. M. O'Boyle , M. Lawlor , H. C. Gallagher , K. J. Murphy , C. M. Regan , Biochem. Pharmacol. 2009, 77, 1254. 19161989 10.1016/j.bcp.2008.12.011

[34] G. Paxinos , K. Franklin , The mouse brain in stereotaxic coordinates, 2nd ed.,Academic Press, New York, 2001.

[35] L. Shen , Z. Liang , H. Yu , Bio Protoc 2017, 7, e2095. 10.21769/BioProtoc.2095 PMC8376565 34458425

[36] N. Chomczynski P Fau‐ Sacchi , N. Sacchi , Anal. Biochem. 1987, 162, 156. 2440339 10.1006/abio.1987.9999

[37] J. R. Wisniewski , A. Zougman , N. Nagaraj , M. Mann , Nat. Methods 2009, 6, 359. 19377485 10.1038/nmeth.1322

[38] J. Rappsilber , M. Mann , Y. Ishihama , Nat. Protoc. 2007, 2, 1896. 17703201 10.1038/nprot.2007.261

[39] S. J. Humphrey , O. Karayel , D. E. James , M. Mann , Nat. Protoc. 2018, 13, 1897. 30190555 10.1038/s41596-018-0014-9

[40] S. Du , S. Xiong , X. Du , T. F. Yuan , B. Peng , Y. Rao , J Vis Exp 2021. 10.3791/62237 33720125

[41] C. Zhang , Y. Chen , B. Sun , L. Wang , Y. Yang , D. Ma , J. Lv , J. Heng , Y. Ding , Y. Xue , X. Lu , W. Xiao , Y. G. Yang , F. Liu , Nature 2017, 549, 273. 28869969 10.1038/nature23883

[42] X. Wang , Z. Lu , A. Gomez , G. C. Hon , Y. Yue , D. Han , Y. Fu , M. Parisien , Q. Dai , G. Jia , B. Ren , T. Pan , C. He , Nature 2014, 505, 117. 24284625 10.1038/nature12730 PMC3877715

