# Supplementary material for: Curcumin Modulates PTPRZ1 Activity and RNA m6A Modifications in Neuroinflammation‐Associated Microglial Response
Source: Adv Sci (Weinh). 2025 Feb 8;12(15):2405263. doi: 10.1002/advs.202405263 (PMC12005744; doi:10.1002/advs.202405263)
Supplement: Supplementary file 1 — Supporting Information [file ADVS-12-2405263-s001.pdf]

ADVANCED SCIENCE
Open Access

Supporting Information

for *Adv. Sci.*, DOI 10.1002/advs.202405263

Curcumin Modulates PTPRZ1 Activity and RNA m6A Modifications in Neuroinflammation-Associated Microglial Response

Ninan Zhang, Ruifan Lin, Wenya Gao, Honglin Xu, Yuejia Li, Xiahe Huang, Yingchun Wang, Xianghong Jing, Wenxiang Meng and Qi Xie**

Curcumin Modulates PTPRZ1 Activity and RNA m6A Modifications in Neuroinflammation-Associated Microglial Response

Ninan Zhang[a,b,c], Ruifan lin[b,c], Wenya Gao[b,c], Honglin Xu[c], Yuejia Li[c], Xiahe Huang[c], Yingchun Wang[c,d,e], Xianghong Jing[a], Wenxiang Meng[c,d,e*] and Qi Xie[f*]

Supporting Information

A

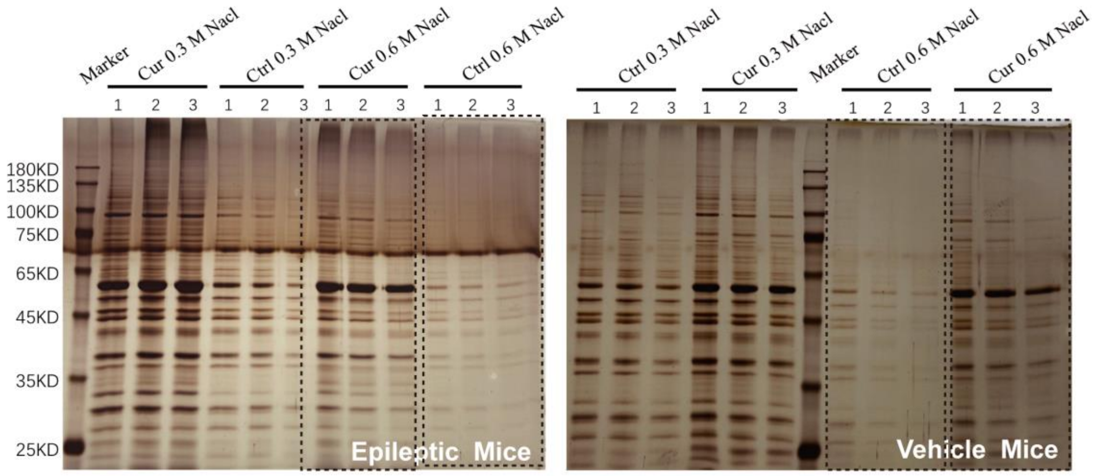

B

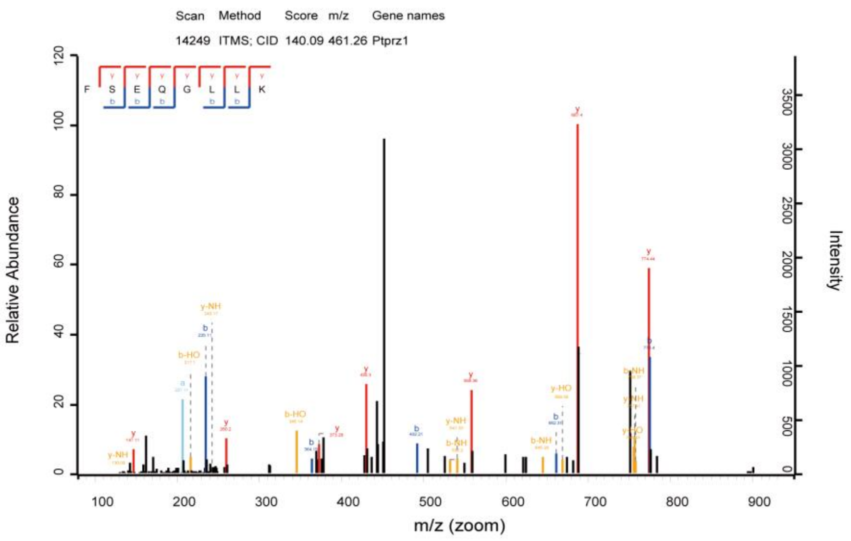

Fig. S1. Identification of curcumin target proteins. (A) Using silver staining to check the target proteins in the 4 experimental groups. The dashed box represents the sample examined by mass spectrometry. (B) The results of mass spectrometry showed the peptide of PTPRZ1.

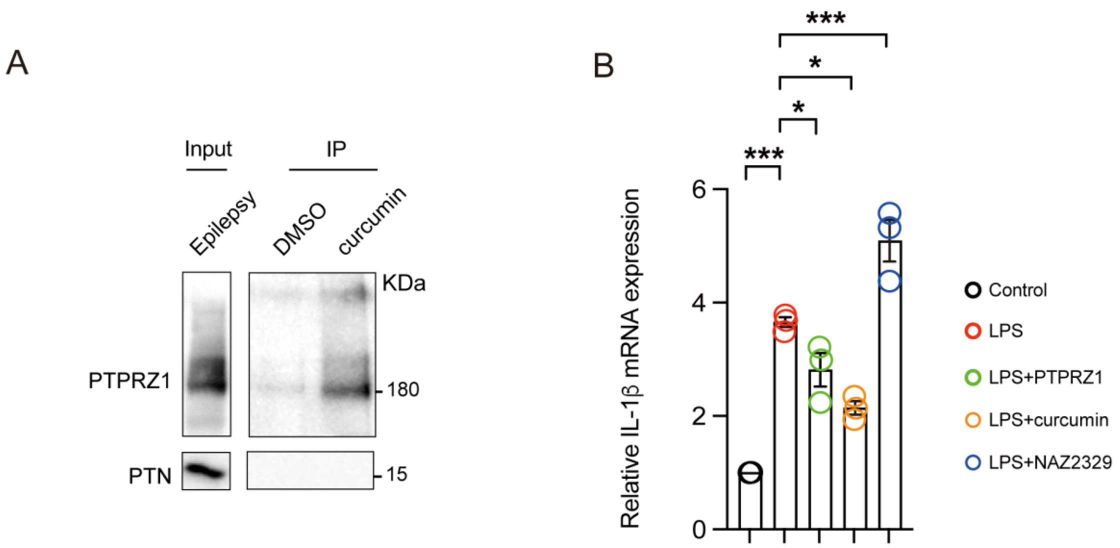

Fig. S2. Curcumin modulation of neuroinflammation may indeed be mediated through its influence on PTPRZ1 activity. (A). Western blot results showed that PTN does not interact with curcumin after status epilepticus. (B) Analysis of mRNA levels of IL-1β. Cells overexpressing PTPRZ1-Flag or pCAH-Flag for 24h, followed by treatment with LPS (250 ng/mL), curcumin (10 μM), NAZ2329 (25 μM), or vehicle (control) for 16h in serum-free medium, were analyzed using qPCR. Data are mean ± SEM., n = 3 biologically independent experiments by Student's *t*-test.

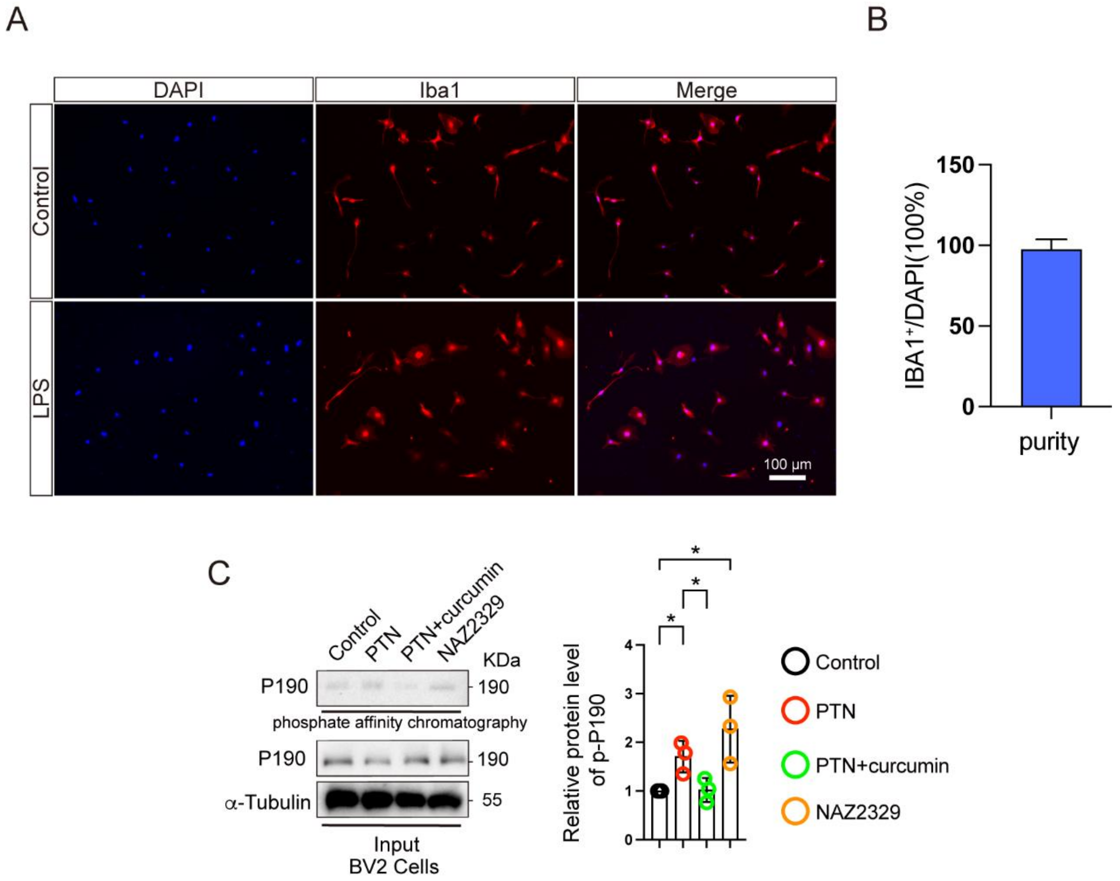

Fig. S3. Reactivity of primary microglia and BV2 cells. (A) Purified primary microglial responds well to LPS exposure. Cells were stained with microglial marker protein Iba1. Scale bar 100 μm. (B) Represents the purity of microglia. (C) Phosphate affinity chromatography using Phos-tag™ agarose in Bv2 cells subjected to various conditions: control, PTN, PTN+curcumin, and NAZ2329. P190 is used as a positive control for PTPRZ1 substrate. Phosphorylated protein quantification of P190. Data are the mean ± SEM. n=3 biologically independent experiments. *$p < 0.05$ by Student's *t*-test.

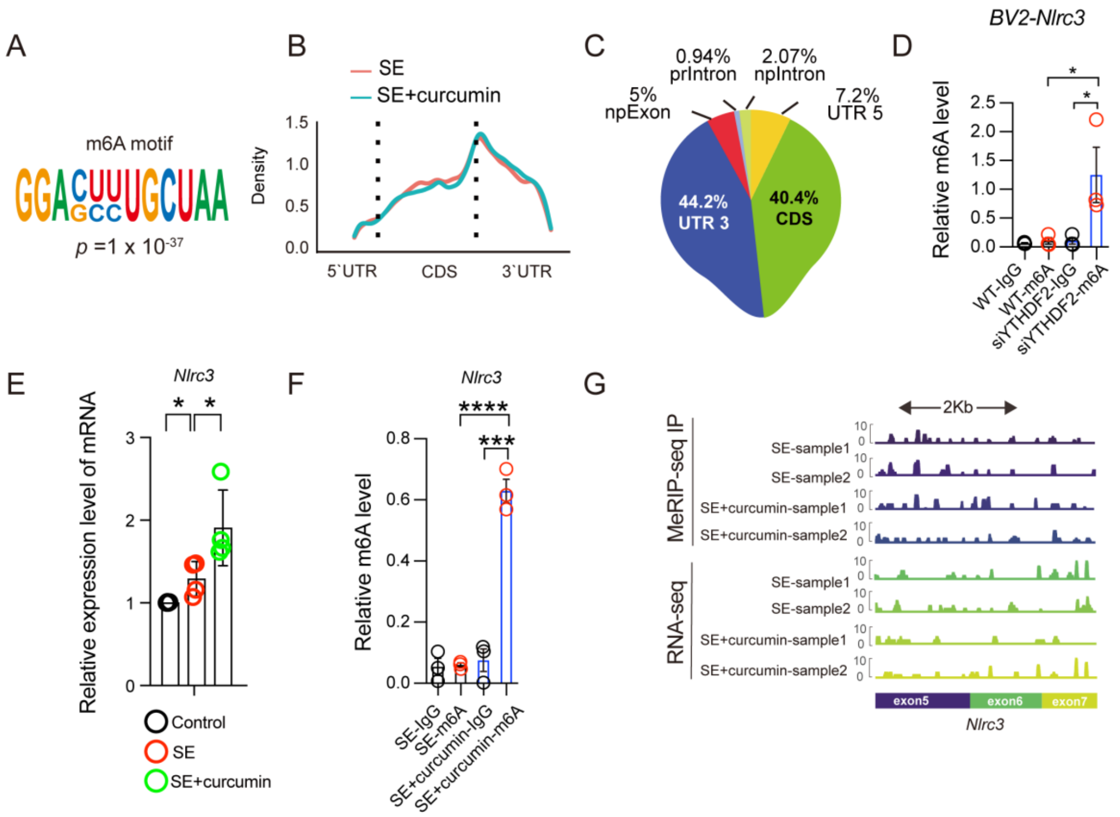

Fig. S4. Curcumin upregulates *Nlrc3* mRNA m6A modification and expression after status epilepticus. (A) Sequence motif identified within m6A peaks by HOMER database. (B) Metagene profiles of m6A peak distribution along a normalized transcript composed of three rescaled non-overlapping segments: 5'UTR, CDS, and 3'UTR in SE and SE + curcumin mice. (C) Pie chart depicting the fraction of m6A peaks in six transcript segments. (D) m6A enrichment in *Nlrc3* in BV2 cells by MeRIP-qPCR. (E) Analysis of *Nlrc3* mRNA levels in control, SE, and SE+curcumin mice groups. Data are mean ± SEM., n = 4 by Student's *t*-test. (F) m6A enrichment in *Nlrc3* between SE and SE+curcumin groups by MeRIP-qPCR. (G) Integrative Genomics Viewer (IGV) tracks displaying MeRIP-seq (upper panels) and RNA-seq (lower panels) read distribution in Nlrc3 mRNA of SE and SE+curcumin mice.

A

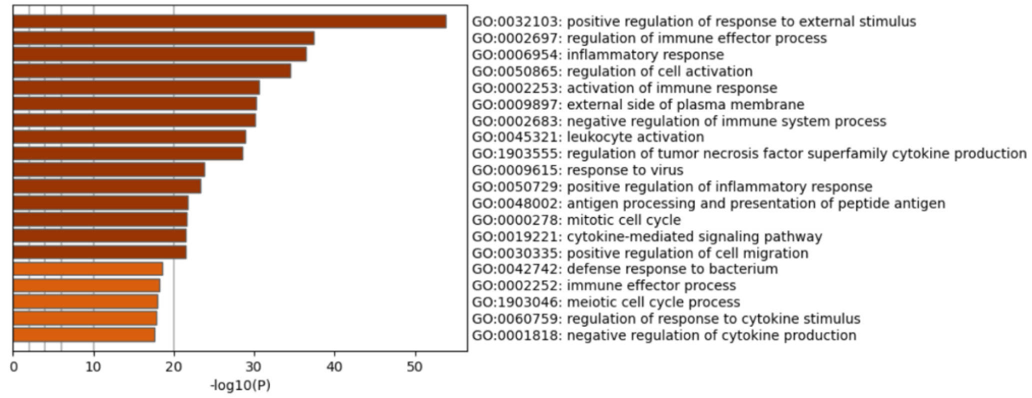

GO analyis for 2234 differential RNA-Seq peaks

B

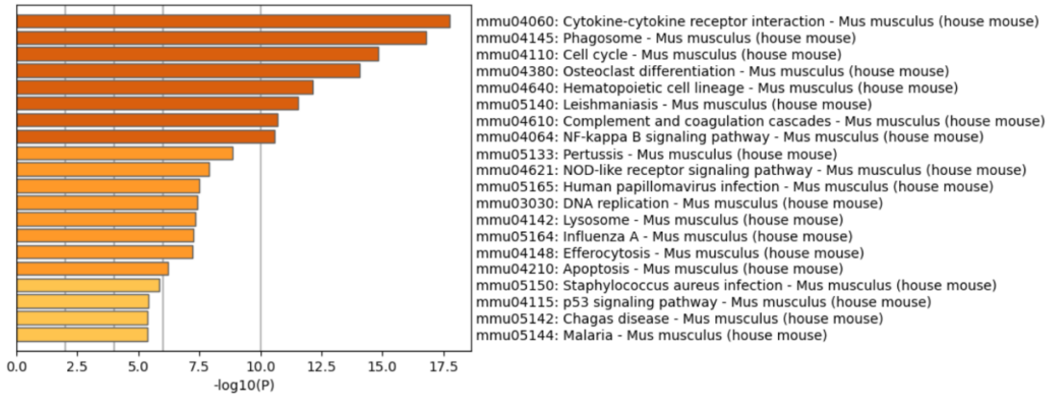

KEGG analysis for 2234 differential RNA-Seq peaks

Fig. S5. The inflammatory response is a critical biological process that can be regulated by curcumin after status epilepticus. (A) GO (Gene Ontology) analysis of 2234 differential genes by RNA-seq in SE with curcumin treatment vs SE groups. (B) KEGG (Kyoto Encyclopedia of Genes and Genomes) analysis of 2234 differential genes by RNA-seq in SE with curcumin treatment vs SE groups.

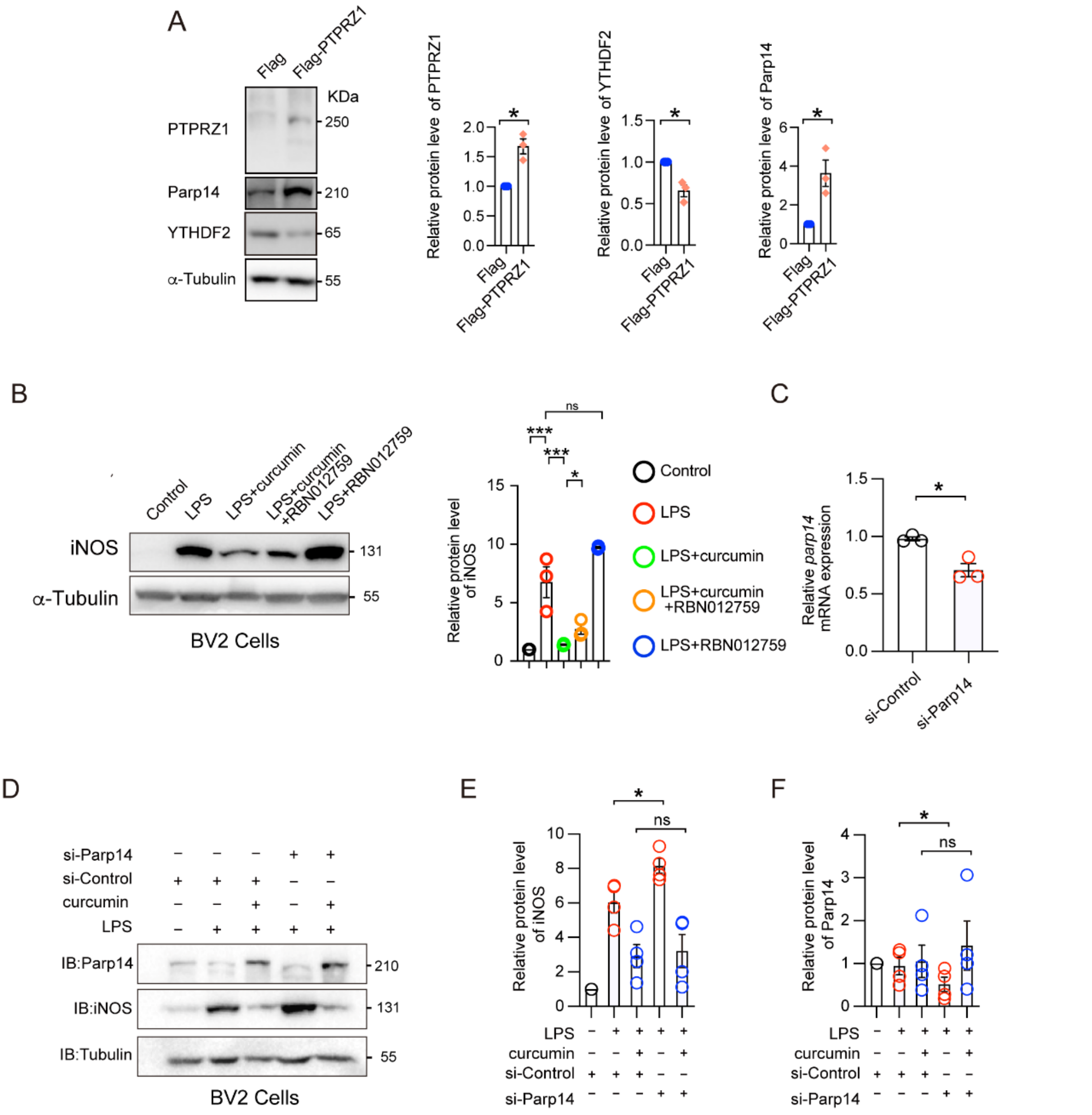

Fig. S6. Parp14 is a critical mediator downstream of curcumin that modulates microglial response. (A) Western blot analysis of PTPRZ1, Parp14, and YTHDF2 in HEK293T cells transfected with either pCAH-Flag vector (control) or pCAH-Flag-PTPRZ1 (overexpression). α-Tubulin is used as a loading control. The protein quantification of PTPRZ1, Parp14, and YTHDF2 after overexpression of PTPRZ1. Data are the mean ± SEM. n=3 biologically independent experiments by Student's *t*-test. (B) Western blot analyses of iNOS expression in BV2 cells treated with LPS (250 ng/mL) with or without curcumin (10 μM) and RBN012759 (1 μM), or vehicle (control) in serum-free medium for 16h. Data are mean ± SEM. n = 3 biologically independent experiments by Student's *t*-test. (C) Real-time qPCR analyses of the knockdown efficiency of *Parp*14 siRNA. (D) Western blotting of iNOS in BV2 cells transfected with Mock (Lipo 2000) or *Parp*14 siRNA and then treated with LPS (250 ng/mL), curcumin (10 μM), or both. (E-F) iNOS and Parp14 expression levels from (D). Data are mean ± SEM., n = 4 biologically independent experiments by Student's *t*-test.

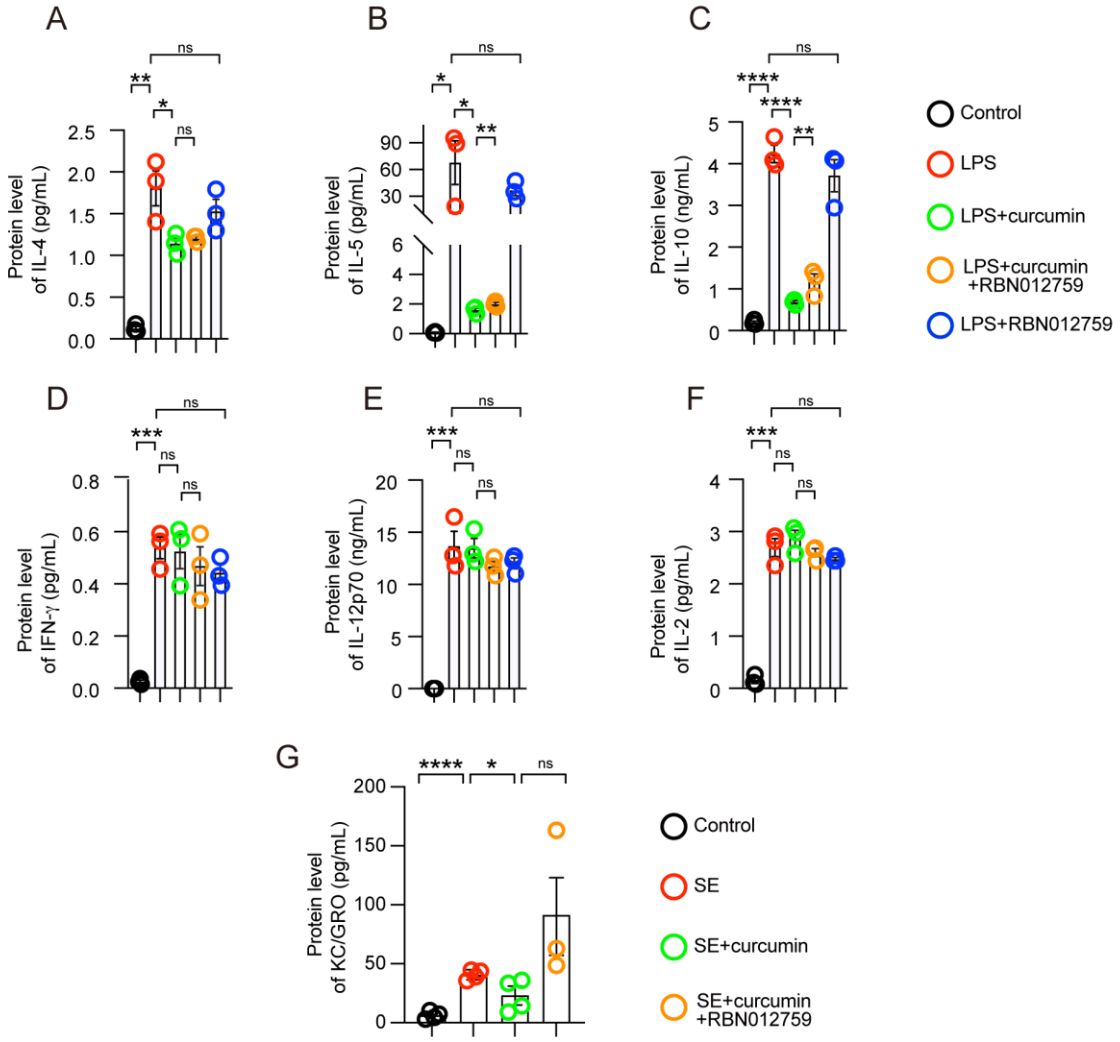

Fig. S7. Multicytokine detection was performed in primary microglia and mice. (A-F) Mouse Proinflammatory V-Plex Tissue Culture Kit analyses of IL-4, IL-5, IL-10, IFN-γ, IL-12p70, and IL-2 expression in primary microglia treated with LPS (250 ng/mL) with or without curcumin (10 μM) and RBN012759 (1 μM), or vehicle (control) for 16h in serum-free medium. Data are mean ± SEM., n = 3 biologically independent experiments by Student's *t*-test. (G) Mouse Proinflammatory V-Plex Tissue Culture Kit analyses of KC/GRO expression in the hippocampus and cortex from different experimental groups of control or SE mice. Data are mean ± SEM., n = 4 or n=3, $*p < 0.05$, $**p < 0.01$, $***p < 0.001$ by Student's *t*-test.
